# Prognostic and Predict Value of Peripheral Blood Circulating Tumor Cells Programmed Death‐Ligand 1 Expression and F‐18‐Fluorodeoxyglucose Metabolic Parameters in Patients With Advanced Non‐Small Cell Lung Cancer Treated With Immune Checkpoint Inhibitors

**DOI:** 10.1002/cam4.72001

**Published:** 2026-06-10

**Authors:** Momo Sun, Aisikaer Aikedan, Zhongying Rui, Enci Ding, Jie Shen

**Affiliations:** ^1^ Department of Nuclear Medicine Tianjin First Central Hospital Tianjin China; ^2^ Nankai University Tianjin China

**Keywords:** ^18^F‐FDG PET/CT, circulating tumor cells, immune checkpoint inhibitor, immunotherapy, non‐small‐cell lung cancer, overall survival, prognosis, response prediction

## Abstract

**Purpose:**

To investigate the concordance and correlation between programmed death‐ligand 1 (PD‐L1) expression in tumor tissue and circulating tumor cells (CTCs), explore the intrinsic link between epithelial‐mesenchymal transition (EMT) and PD‐L1 expression in CTCs, and examine the predictive value of FDG metabolic parameters, CTCs and their PD‐L1 expression for early tumor response and long‐term prognosis in patients with advanced non‐small cell lung cancer (NSCLC) receiving immune checkpoint inhibitor (ICI) monotherapy or ICI combined with chemotherapy as first‐line treatment.

**Methods:**

A total of 42 patients with advanced or metastatic NSCLC who received ICI monotherapy or combination chemotherapy as first‐line treatment were enrolled as the study population. Pre‐treatment peripheral blood CTCs and PD‐L1 expression on CTCs were detected. Furthermore, F‐18‐fluorodeoxyglucose Positron Emission Tomography/Computed Tomography (18F‐FDG PET/CT) tumor metabolic parameters, including maximum standardized uptake value (SUVmax), standard uptake value of lean body mass (SUL), metabolic tumor volume (MTV), total lesion glycolysis (TLG), whole‐body metabolic tumor volume (WMTV), and whole‐body total lesion glycolysis (WTLG) were collected and measured. Tumor early response was monitored using response evaluation criteria in solid tumors (RECIST criteria) at 8 weeks after ICI treatment, and the differences in CTC PD‐L1 expression, FDG metabolic parameters, and clinical data between early respongders and non‐responders were analyzed. Finally, the enrolled patients were followed up to evaluate progression‐free survival/overall survival (PFS/OS) and its associated predictive factors. Bootstrap internal validation (1000 repeated samples) was used to evaluate the robustness of the above prognostic model.

**Results:**

The percentage of PD‐L1‐positive cells in tumor tissue and CTC represents different immune characteristics of patients, and the correlation between these two percentages was not statistically significant (*r* = 0.041, *p* = 0.827). However, an intrinsic connection was identified between the EMT process and PD‐L1 expression in CTCs, with PD‐L1 expression in CTCs gradually increasing as the EMT process in CTCs changed. Furthermore, tumor proportion score (TPS) demonstrated a significant positive correlation with SUVmax (*r* = 0.684；*p* < 0.001) and SUL (*r* = 0.603；*p* < 0.01), with SUVmax and SUL increasing as TPS increased. Patients who exhibited an early response after 8 weeks of treatment had significantly higher SUVmax and SUL values, but lower mixed CTCs, than those in the non‐response group (*p* < 0.05). The combination of SUVmax and PD‐L1+ mixed CTCs yields a sensitivity of 81% and a specificity of 71% for predicting early response to ICI therapy in patients with NSCLC, but the combination did not result in a substantial enhancement in predictive efficacy. PD‐L1+ mesenchymal CTCs (HR = 5.520, 95% CI 1.993–15.291) and WTLG (HR = 4.315, 95% CI 1.864–9.991) were identified as independent predictors of PFS, while PD‐L1+ mesenchymal CTCs (HR = 2.880, 95% CI 1.124–7.381) were identified as independent predictors of OS. Bootstrap resampling analysis (*B* = 1000 iterations) revealed that PD‐L1+ mesenchymal CTCs and WTLG achieve an apparent area under the ROC curve (AUC) of 0.822 for predicting PFS, with a bootstrap‐corrected mean AUC of 0.821 (95% CI: 0.714–0.917). For OS prediction, PD‐L1+ mesenchymal CTCs yielded an apparent AUC of 0.893 and a bootstrap‐validated mean AUC of 0.88 (95% CI: 0.655–0.975). Furthermore, the combination of PD‐L1+ mesenchymal CTCs and WTLG revealed that patients with WTLG < 1627.4 (g) and PD‐L1+ mesenchymal CTCs < 1/5 mL exhibited prolonged PFS and OS (median PFS was 12.5 months; median OS was 18.5 months), while those with WTLG ≥ 1627.4 (g) and PD‐L1+ mesenchymal CTCs ≥ 1/5 mL experienced the shortest PFS and OS (median PFS was 1.35 months; median OS was 3.0 months). Patients with PD‐L1+ mesenchymal CTCs ≥ 1/5 mL or WTGL ≥ 1627.4 (g) exhibited a PFS and OS that fell between the two groups (median PFS was 6.0 months; median OS was 8.0 months). Stability analysis was conducted on the prognostic model after the combination of PD‐L1+ mesenchymal CTCs and WTLG. Bootstrap resampling analysis (*B* = 1000 iterations) showed that the AUC value of the original ROC curve was 0.821, the mean AUC was 0.823 (95% CI: 0.716–0.914) when predicting PFS. For OS prediction, the AUC value of the original ROC curve was 0.875, the mean AUC was 0.874 (95% CI: 0.751–0.966).

**Conclusion:**

EMT is associated with PD‐L1 expression in CTCs, with CTCs exhibiting a mesenchymal phenotype tending to have higher PD‐L1 expression. PD‐L1+ mixed CTCs, SUVmax, and SUL were associated with early response to ICI‐based treatment. Preliminarily, PD‐L1+ mesenchymal CTCs, as an exploratory biomarker, were significantly associated with PFS and OS and may provide prognostic information complementary to conventional tissue‐based PD‐L1 assessment and FDG metabolic parameters. However, because PD‐L1 expression in primary tumor tissue and CTCs was assessed using different detection methodologies, the observed discordance should be interpreted as potentially reflecting both biological heterogeneity and methodological variation. These findings require further validation in larger cohorts using harmonized detection platforms.

## 1 Introduction

Notwithstanding ongoing exploration and enhancement in pharmaceuticals and therapeutic modalities for non‐small cell lung cancer (NSCLC), the 5‐year survival rate for NSCLC patients remains low. In recent years, with the advent of tumor immunotherapy, immune checkpoint inhibitors (ICIs) have emerged as a pivotal therapeutic modality for advanced NSCLC, providing substantial benefits to a considerable proportion of patients by virtue of their capacity to elicit an immune response [1]. However, studies have shown that only a small proportion of NSCLC patients are sensitive to ICI therapy [2, 3], and the response rate of unselected NSCLC patients receiving ICI monotherapy is typically less than 20% [4]. Consequently, the next and more challenging hurdle is to extend the benefits of ICI therapy to a more suitable population. In order to enhance the therapeutic efficacy of ICIs, it is imperative that researchers identify appropriate biomarkers to accurately assess and predict the treatment response of NSCLC patients to ICIs. Among the numerous biomarkers under investigation, programmed death‐ligand 1 (PD‐L1) expression level is widely considered an important indicator for evaluating ICI efficacy. The majority of studies have focused on PD‐L1 expression in primary tumor tissue, attempting to establish it as a standard for predicting treatment outcomes. While PD‐L1‐positive tumor patients have been observed to demonstrate superior treatment responses in certain cases, a significant proportion of PD‐L1‐positive tumor patients do not respond to ICI therapy.

The use of F‐18‐fluorodeoxyglucose Positron Emission Tomography/Computed Tomography (18F‐FDG PET/CT) and circulating tumor cell (CTC) detection in NSCLC immunotherapy has distinct advantages and limitations. 18F‐FDG PET/CT has been extensively applied in oncology, providing supplementary functional information in comparison to conventional imaging modalities that depend on morphological alterations. Studies have shown that semi‐quantitative parameters based on FDG metabolism can reflect the immune cell status within the tumor microenvironment, thereby being applied to predict response to ICI therapy [5, 6, 7, 8, 9, 10] and for prognostic evaluation [11, 12, 13]. However, the complexity of the tumor microenvironment and the involvement of stromal cells have the potential to delay the efficacy of immunotherapy, and the early FDG uptake parameters may not accurately predict long‐term efficacy. Furthermore, there may be significant variations in immune cell infiltration and metabolic activity across different regions of the tumor, which may hinder the comprehensive reflection of this complexity by FDG metabolic parameters. Liquid biopsy techniques, exemplified by the presence of CTCs, provide a non‐invasive approach for patient screening, treatment monitoring, and assessment of treatment response [14, 15, 16, 17, 18, 19, 20] and prognosis [21, 22, 23, 24, 25, 26]. Although studies have confirmed an intrinsic link between the epithelial‐mesenchymal transition (EMT) in tumor tissues and PD‐L1 expression, the relationship between different CTC subtypes and their corresponding PD‐L1 expression status remains unclear. The potential of different CTC subtypes and their PD‐L1 expression to predict the PD‐L1 status of the primary tumor and early response to ICI therapy, as well as their prognostic value in ICI‐treated patients, remains to be explored. Furthermore, preliminary studies have combined FDG metabolic parameters with CTC for the diagnosis and prognosis evaluation in NSCLC, and have shown potential value. However, the limited number of available studies, coupled with discrepancies in selected semi‐quantitative metabolic parameters and variations in CTC biomarkers and isolation methodologies, have precluded consistent conclusions. This leads to further exploration of the value of the combined application of the two methods.

Given the respective advantages and limitations of PET and CTC, the objective of this study was to investigate the concordance and correlation between PD‐L1 expression of tumor tissue and CTCs in patients with advanced or metastatic NSCLC receiving ICI monotherapy or ICI‐chemotherapy combinations. Preliminary delve into the intrinsic relationship between the EMT process in CTCs and PD‐L1 expression. Evaluate the predictive value of FDG metabolic parameters combined with CTC PD‐L1 expression for early tumor response to ICI therapy, and their prognostic significance for survival outcomes.

## 2 Methods

### 2.1 Patient Population

This study is a single‐centre, observational study that enrolled 42 patients newly diagnosed with stage III‐IV NSCLC who were planned to undergo ICI therapy. Prior to the commencement of treatment, all patients underwent 18F‐FDG PET/CT scans and peripheral blood CTC detection. The inclusion criteria were as follows: (1) treatment‐naïve patients with advanced NSCLC at stage III‐IV. (2) Patients with no history of other malignant tumors or cancers. (3) Patients with subsequent pathological examination of lung lesions, including only adenocarcinoma and squamous cell carcinoma, excluding other NSCLC subtypes. The exclusion criteria included: (1) a history of other types of cancer; (2) an interval of more than 2 weeks between PET/CT examination and pathological diagnosis; (3) coagulation of peripheral blood samples; (4) hemolysis of peripheral blood samples; (5) peripheral blood sample volume less than 5 mL. Following enrolment, clinical and pathological information for each patient was recorded.

All procedures of this study were approved by the Ethics Committee of Tianjin First Central Hospital and conducted in accordance with the 1964 Declaration of Helsinki and its subsequent amendments or similar ethical standards. Informed consent was obtained from all participants. Peripheral venous blood was collected from all patients using vacuum blood collection tubes on the same day before the PET/CT scan. CTC enrichment was completed within 24 h of peripheral blood collection.

### 2.2 18F‐FDG PET/CT Scan

18F‐FDG PET/CT scans were performed in strict accordance with the European Association of Nuclear Medicine (EANM) guidelines. The PET/CT scans were conducted using a biograph mCT‐S64 PET/CT (Siemens Healthineers, Erlangen, Germany) with an axial field of view of 21.6 cm. The radiotracer used is 18F‐FDG, with a radiochemical purity greater than 95%. Prior to undergoing the imaging procedure, patients were required to fast for a period of 6 h (with a target blood glucose level of ≤ 8 mmol/L for diabetic patients and ≤ 7 mmol/L for non‐diabetic patients receiving 18F‐FDG). All patients were instructed to discontinue glucocorticoids for at least 72 h and complete antibiotic treatment for ≥ 3 days before PET/CT imaging to avoid interference with FDG uptake. Patients with unavoidable glucocorticoid use were recorded and adjusted in statistical analysis. The radiotracer was administered intravenously at a dose of 0.12 mCi per kilogram of body weight, and image acquisition was performed approximately 60 min later. Each patient underwent 6–7 bed positions from head to mid‐thigh, with a scan time of 2.5 min per bed position. CT scans were performed on the same scanner without the need for oral or intravenous contrast agents. The CT images were used for attenuation correction of the PET scans, with the following scanning parameters: 120 kV, 101 mA, gantry rotation time of 0.5 s, and all CT images were acquired with a slice thickness of 5 mm. The PET images were then reconstructed iteratively, employing a 200 × 200 pixel matrix size. Finally, the PET and CT images were transferred to a workstation for fusion analysis.

### 2.3 PET/CT Imaging Analysis

The interpretation of PET/CT images was conducted by two experienced nuclear medicine physicians with more than 5 years of clinical experience, who were blinded to the patients' clinical information, pathological results and treatment outcomes to avoid subjective bias. In instances of discrepancy, a consensus was reached through mutual discussion. The analysis of PET metabolic parameters, encompassing maximum standardized uptake value (SUVmax), standard uptake value of lean body mass (SUL), metabolic tumor volume (MTV), total lesion glycolysis (TLG), whole‐body metabolic tumor volume (WMTV), and whole‐body total lesion glycolysis (WTLG), was conducted utilizing the TrueD software. For each target lesion, a volume of interest (VOI) was defined by applying an absolute SUV threshold of 2.5, a classic and widely validated standard for 18F‐FDG PET/CT tumor metabolic parameter analysis in NSCLC research [5, 7, 12]. This threshold was selected for its broad application in existing literature and its ability to effectively discriminate FDG‐avid NSCLC tissue from physiological uptake in treatment‐naive advanced NSCLC patients with distinct tumor metabolic activity. Manual correction was applied to exclude physiological uptake regions and necrotic areas with CT density 0 HU, minimizing inflammatory uptake interference. Adaptive threshold methods were evaluated but not adopted: the 41% SUVmax threshold was deemed unstable due to its reliance on SUVmax (susceptible to tumor necrosis, cystic degeneration, or local inflammation) and complexity in handling multiple metastases; the liver baseline threshold (mean liver SUV ± 2SD) was considered inappropriate for moderate‐to‐high metabolic NSCLC, as it may overexpand lesion boundaries and increase variability due to individual differences in liver metabolism. Necrotic areas (CT density < 10 HU) were carefully masked. SUVmax was defined as the highest single‐pixel SUV within the lesion. SUL values were corrected for lean body mass using sex‐specific Janmahasatian equations [27]. MTV was segmented using a fixed SUV threshold of 2.5, with volumes < 1 cm^3^ excluded. The calculation of TLG was determined by the multiplication of mean standardized uptake value and MTV. Whole‐body indices (WMTV and WTLG) represented the sum of all target lesions.

To assess inter‐reader variability for SUVmax, SUL, MTV, WMTV, TLG and WTLG measurements, the intraclass correlation coefficient (ICC) was calculated between the two physicians. 15 patients were randomly selected, and the results from the two‐way mixed model demonstrated that the ICC value for SUVmax measurement was 0.98 (95% CI: 0.86–0.99), for SUL was 0.95 (95% CI: 0.80–0.97), for MTV was 0.89 (95% CI: 0.75–0.92), for TLG was 0.88 (95% CI: 0.70–0.95), for WTMV was 0.77 (95% CI: 0.60–0.93), and for WTLG was 0.75 (95% CI: 0.58–0.92). These results all indicated high consistency in the measurements obtained by the two radiologists, with minimal inter‐rater variability that would not compromise the reliability of the findings of this study. For the three cases with initial discrepancies, a consensus was reached through joint re‐evaluation of images, combining CT morphological features and clinical data to ensure the accuracy of metabolic parameter measurements.

#### 2.3.1 CTC Isolation, Enumeration, and PD‐L1 Expression Detection

**FIGURE 1:**
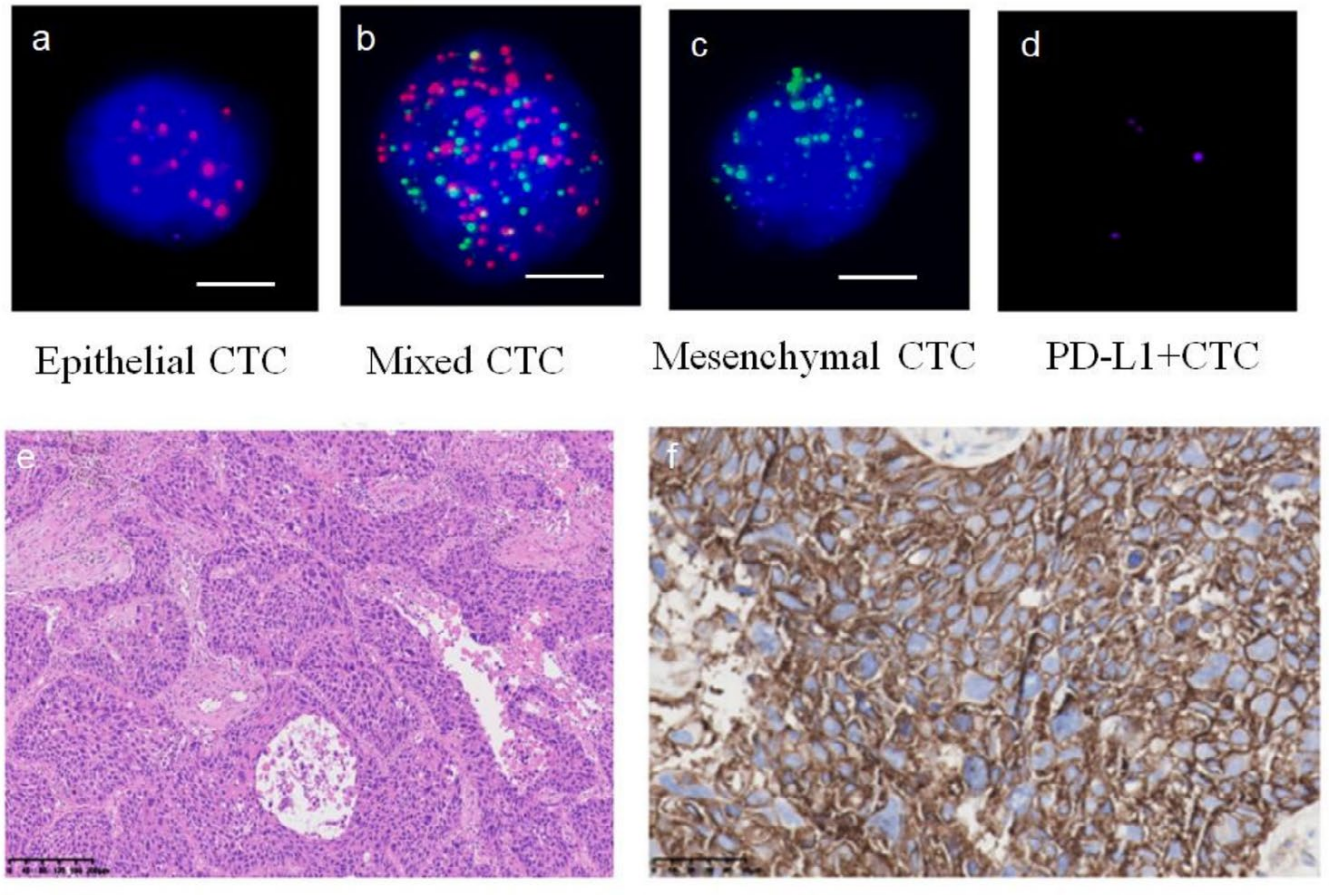
Representative images of CTC and immunohistochemical analysis results. (a–d) Epithelial‐mesenchymal transition phenotype and PD‐L1 positive phenotype of CTCs determined by RNA in situ hybridization. Nuclei are stained with DAPI (blue); (a) Epithelial CTCs are stained with epithelial cell adhesion molecule and cytokeratin 8/18/19 markers (red dots); (b) Mixed CTCs contain both epithelial (red) and mesenchymal (green) markers; (c) Mesenchymal CTCs are stained with Vimentin and Twist markers (green dots); (d) PD‐L1 positive CTCs are stained with PD‐L1 marker (purple). Scale bar, 5μm. Representative images of (e) Hematoxylin and Eosin staining and (f) PD‐L1 immunohistochemistry in tumor tissue (approximately 95% of tumor cells are PD‐L1 positive). Scale bar, 100μm. CTC, circulating tumor cell; PD‐L1, programmed death‐ligand 1.

This study employs a multi‐probe immunofluorescence morphology method based on the CanPatrol platform to quantify CTCs and perform subtype analysis. The performance of the CanPatrol platform in such tumors has been validated by several clinical studies [28, 29, 30, 31, 32], demonstrating stable detection performance in NSCLC. Tumor cell recovery rate: When 10–200 HepG2 cells were spiked into 5 mL of peripheral blood, the average recovery rate ranged from 80% to 89% with a linear correlation coefficient (*R* ^2^) of 0.999, ensuring the stable detection of CTCs at low concentrations. Leukocyte depletion efficiency: Reaching 99.98%, the platform adopts 8 μm nanomembrane filtration combined with red blood cell lysis technology to reduce background interference and improve specificity. Subtype detection capability: It can stably distinguish epithelial, mixed, and mesenchymal CTCs. The detection rate of mixed CTCs in NSCLC is 75.3%, and the detection rate of mesenchymal CTCs in metastatic NSCLC is 65.8%, both significantly higher than that in the benign disease group (0%). Diagnostic performance: For distinguishing NSCLC from benign diseases, the sensitivity and specificity of total CTCs are 81.6% and 86.8%, respectively; the mixed CTCs achieve a specificity of 100% (no false positives in benign diseases) with a cutoff value of 1 cell/5 mL; for predicting distant metastasis in NSCLC, the mesenchymal CTCs have a sensitivity of 65.85% and a specificity of 75.0%. It is suitable for detecting EMT‐phenotypic CTCs across multiple cancer types, providing reliable support for clinical diagnosis and metastatic risk assessment. Peripheral blood samples (5 mL) were collected using ethylenediaminetetraacetic acid (EDTA) anticoagulant tubes, and the blood samples were processed within 4 h. Initially, the blood samples were transferred to cell preservation tubes to lyse red blood cells. Following a 30‐min incubation at room temperature, the samples were subjected to a centrifugal process at 600 × g for 5 min, after which the resultant pellet was filtered through a nanoporous membrane with an 8‐μm pore size. Following enrichment on the nanoporous membrane, the cells were subjected to RNA fluorescence in situ mixedization using six probes, including cytokeratin 8 (CK8), cytokeratin 18 (CK18), cytokeratin 19 (CK19), epithelial cell adhesion molecule (Epcam), vimentin and cluster of differentiation 45 (CD45) at 40°C. Subsequently, pre‐amplification, amplification, and signal mixedization reactions were performed at 40°C to achieve multi‐level amplification of the fluorescence signal. Following 4′,6‐Diamidino‐2‐Phenylindole (DAPI) staining, images were scanned and the results were interpreted and statistically analyzed using an automated fluorescence microscope. The criteria for CTC determination were as follows: a clear nuclear morphology and a distinct, uneven nuclear staining pattern were observed after DAPI staining. The expression of signal points was used to classify CTCs into epithelial CTCs (CK8/18/19/Epcam+, Vimentin−, CD45−), mixed CTCs (CK8/18/19/Epcam/Vimentin+, CD45−), and mesenchymal CTCs (Vimentin+, CK8/18/19/Epcam−, CD45−). Subsequently, the expression level of PD‐L1 Messenger RNA (mRNA) in CTCs was detected by RNA in situ hybridization (RNA‐ISH). Capture probes were designed to capture specific PD‐L1 mRNA, and then conjugated with branched DNA (bDNA) signal amplification probes to create a branched structure. Finally, probes labeled with fluorescent dyes were mixedized with the bDNA sequence. PD‐L1 positivity is defined based on the number of RNA‐ISH signal dots (≥ 4 dots/cell) and proportion (≥ 1% of total viable tumor cells). The counting process adopts a two‐step method of automated preliminary screening combined with manual review, ensuring the accurate classification of CTC subtypes. The results were then subjected to analysis using a fluorescence microscope (Figure 1a–d).

### 2.4 PD‐L1 Expression Detection in Tumor Tissue

Prior to the treatment, PD‐L1 staining was performed on tumor tissue from all patients. All primary tumor tissue specimens were collected by fine needle biopsy (FNB). The detailed procedures were as follows: All biopsies were performed under CT guidance. Preoperative CT scanning was applied to confirm the location, size, and adjacent anatomical relationship of primary tumors, so as to determine the optimal puncture route and avoid vital structures such as large blood vessels and bronchi. Appropriately sized biopsy needles were used to accurately puncture into primary tumor lesions under real‐time CT monitoring. The harvested tissue samples were immediately fixed in 10% neutral formalin solution and subsequently processed into formalin‐fixed paraffin‐embedded (FFPE) specimens. The proportion of tumor cells in all qualified specimens was ensured to be no less than 50% to meet the criteria for PD‐L1 detection. The assessment of PD‐L1 expression in tumor cells was conducted using an anti‐PD‐L1 rabbit monoclonal antibody on 4 μm FFPE tissue sections. Slides were processed on an Autostainer Link 48 (Dako AS480, Glostrup Kommune, Denmark) using a validated automated staining protocol for PD‐L1 IHC with an anti‐PD‐L1 22C3 primary antibody (Dako). All slides were evaluated under an inverted TS100 microscope at a magnification of 9200 (Nikon Corporation, Tokyo, Japan). In accordance with the tumor proportion score (TPS) scoring principle, only partial or complete membranous staining of viable tumor cells was considered positive for tissue PD‐L1 assessment; cytoplasmic staining and staining of immune or stromal cells were not included in TPS calculation. TPS was calculated as (number of PD‐L1‐stained positive tumor cells/total number of viable tumor cells in the sample) × 100% (Figure 1e,f).

#### 2.4.1 Efficacy Evaluation of ICI Treatment

The follow‐up period extended from 1 October 2022 to 30 December 2024. The median follow‐up time for patients enrolled in the study was 13.5 months (range, 1.3–24 months). The evaluation of efficacy was conducted in accordance with the response evaluation criteria in solid tumors (RECIST criteria) version 1.1 [33] protocol. The definition of early tumor response was the response of lesions within 8 weeks of the commencement of treatment. Patients with stable disease (SD), progressive disease (PD), and those with unevaluable response due to early death were considered to have no early tumor response (*n* = 22, 52%), while patients with partial response (PR) or complete response (CR) were considered to have an early tumor response (*n* = 20, 48%).

#### 2.4.2 Variables Assessed for Survival Outcomes

Progression‐free survival (PFS) was defined as the time from the commencement of immunotherapy to progression/death from any cause. Overall survival (OS) was defined as the time from the commencement of immunotherapy to death from any cause. During the follow‐up period, 33 (79%) NSCLC patients experienced progression and 31 (74%) died.

### 2.5 Statistical Analysis

The data collected in this study were processed and analyzed using PASS 15.0, SPSS 27.0 and R 4.4.0 software. Count data are expressed as the number of cases (*n*), and categorical data are expressed as the number of cases and percentage (%). The normality of continuous variables was assessed using the Shapiro–Wilk test. Continuous variables that conformed to a normal distribution were described as mean ± standard deviation, whilst non‐normally distributed variables were represented by median (interquartile range). (1) Previous studies [34, 35, 36, 37] reported 24‐month mortality rates of 50%–80% in NSCLC patients following ICI treatment (monotherapy or combined with chemotherapy). Using a reference mortality probability of 60% [37] from comparable cohorts with similar patient characteristics and therapeutic approaches, and further informed by analogous findings from Zhang et al. [38], we performed Cox regression analysis to evaluate CTCs and SUV metabolic parameters as predictors of mortality. The regression coefficient (β) for the primary independent variable was 1.55 with an estimated standard deviation of 0.49. Sample size calculation was conducted using PASS 15.0 software (NCSS LLC) for Cox regression modeling, with a two‐sided α level of 0.05 and power (1 − β) of 0.9. This yielded a requirement of 64 patients. Accounting for an anticipated 20% dropout rate, the minimum target enrollment was set at 77 patients to ensure scientific rigor. However, due to strict eligibility criteria and study time constraints, the final analyzed cohort comprised 42 eligible patients. (2) The Wilcoxon signed‐rank test was employed for the purpose of making comparisons between paired and non‐normally distributed data. The independent samples *t*‐test was utilized for the purpose of making intergroup comparisons between two groups of normally distributed data. The one‐way ANOVA was employed for the purpose of making comparisons among multiple groups, and non‐parametric rank‐sum tests were used for the purpose of making comparisons between two or more groups of non‐normally distributed data. The chi‐square test or Fisher's exact test was used to compare categorical variables, and the Pearson chi‐square test was used to compare rates among multiple groups. (3) Spearman's rank correlation was used for correlation analysis of the data. (4) Logistic regression was used to screen for independent influencing factors of PD‐L1 positivity in primary tumors. (5) ROC curve analysis was performed on SUVmax, SUL, PD‐L1+ mixed CTCs, and the combination of SUVmax and PD‐L1+ mixed CTCs to evaluate the predictive efficacy of the above influencing factors on early tumor response to ICI therapy in patients. The sensitivity, specificity, and area under the curve (AUC) of the aforementioned factors were calculated, and the Delong test was used to compare the diagnostic efficacy of each ROC curve. (6) Cox regression analysis was used to construct univariate and multivariate models for PFS and OS to screen for independent influencing factors related to prognosis. Variables with *p* < 0.10 in univariate analysis were considered for inclusion in the multivariable Cox proportional hazards model. Given the sample size, only the most significant and clinically relevant variables were retained in the final multivariable model to minimize overfitting. Kaplan–Meier analysis was used to assess PFS and OS in all patients and specific subgroups, and the results are expressed as median (M) in months with a 95% confidence interval (CI). The log‐rank test was used to statistically compare Kaplan–Meier curves. For PD‐L1+ mesenchymal CTCs and WTLG in the combined prognostic model, given the current lack of well‐defined clinical cut‐off criteria, the median split method was adopted in this study to determine the stratification cut‐offs. As a robust and commonly used strategy in exploratory prognostic research [39, 40, 41, 42], this approach can effectively balance subgroup distribution and capture clinical heterogeneity without introducing arbitrary thresholds. A *p*‐value of less than 0.05 was considered to be statistically significant. The generation of graphs and curves was facilitated by the use of GRAPHIPAD PRISM 9.1. (7) To assess the internal validity and potential overfitting of the prognostic model, the bootstrap internal validation with 1000 iterations was performed by the use of R 4.4.0.

## 3 Results

### 3.1 Patient Characteristics

42 treatment‐naïve stage III–IV NSCLC patients were enrolled (mean age 68; 29 male/13 female). Most (*n* = 37, 88%) received a combination of anti‐PD‐1/PD‐L1 therapy and chemotherapy. Baseline characteristics are summarized in Table 1.

**TABLE 1:** Patients' characteristics.

Characteristic		Subgroup	*n*	Mean/median	Range
Clinical data	Age (years)	< 63	10 (24%)	—	47–89
		≥ 63	32 (76%)	—	—
	Gender	Female	29 (69%)	—	—
		Male	13 (31%)	—	—
	ECOG score	0	19 (45%)	—	—
		1	22 (52%)	—	—
		2+	1 (2%)	—	—
	Smoking	Smoker	27 (64%)	—	—
		No smoker	15 (36%)	—	—
	Site of primary tumor	Left lung	20 (48%)	—	—
		Right lung	22 (52%)	—	—
	Diameter max	—	—	5.45 ± 2.42	1.2–10.79
	TNM stage (AJCC 9th ed.)	III	24 (57%)	—	—
		IV	18 (43%)	—	—
	Distribution of metastatic sites	Regional lymph node	24 (57%)	—	—
		Distant lymph node	11 (26%)		
		Bone	9 (21%)	—	—
		Liver	6 (14%)	—	—
		Pleural	4 (10%)	—	—
		Adrenal gland	3 (7%)	—	—
		≥ 2 sites of distant metastasis	12 (29%)	—	—
	Histology	AC	18 (43%)	—	—
		SCC	24 (57%)	—	—
	PD‐L1 TPS	< 1%	21 (50%)	—	—
		1%–49%	14 (33%)	—	—
		≥ 50%	7 (17%)	—	—
CTC data	Total CTCs	—	—	9	1–50
	Epithelial CTCs/5 mL	—	—	4	0–15
	Mixed CTCs/5 mL	—	—	3	0–10
	Mesenchymal CTCs/5 mL	—	—	0	0–25
	PD‐L1 + CTCs/5 mL	—	—	5	0–25
	PD‐L1 + Epithelial CTCs/5 mL	—	—	2	0–8
	PD‐L1 + Mixed CTCs/5 mL	—	—	1	0–5
	PD‐L1+ Mesenchymal CTCs/5 mL	—	—	0	0–20
	PD‐L1 + CTC (%)	—	—	56	0–100
FDG PET data	SUVmax	—	—	19.39 ± 7.34	3.86–34.50
	SUL	—	—	14.76 ± 5.49	3.50–25.70
	MTV (mm^3^)	—	—	97.76	8.56–654.59
	WMTV (mm^3^)	—	—	253.39	30.98–1172.98
	TLG (g)	—	—	643.28	20.43–4693.75
	WTLG (g)	—	—	1627.38	104.65–47,370.20
Treatment	Immunotherapy combine with chemotherapy	—	37 (88%)	—	—
	Immunotherapy	—	5 (12%)	—	—
Early response	Response	—	20 (48%)		
	No response	—	22 (52%)		

### 3.2 Correlation Between PD‐L1 Expression in CTCs and Tumor Tissue

CTCs were detected in all 42 NSCLC patients. The number of CTCs in patients, as well as the expression of PD‐L1 in each subtype, are detailed in Table 1. The detection rate of PD‐L1 in CTCs was found to be 98%. Furthermore, 31 patients exhibited PD‐L1+ CTCs ≥ 3/5 mL, accounting for 74% of the entire patient population. PD‐L1 expression was observed in tumor tissue in 21 out of 42 (50%) patients. In all patients, only one case showed no PD‐L1 expression in both CTCs and tumor tissue. Conversely, in the remaining 19 cases (45%) where negative PD‐L1 expression was observed in tumor tissue, PD‐L1 expression was detected in CTCs.

The Wilcoxon signed‐rank test demonstrated that the TPS was significantly lower than CTC PD‐L1+ percentage (median: 0.5% vs. 56%; *p* < 0.001; Figure S1a), with no correlation (*r* = 0.03, *p* = 0.87; Figure S2a). Because it is difficult to calculate the percentage of PD‐L1+ CTCs when the number of CTCs is extremely low, we further analyzed the correlation of PD‐L1 expression between CTCs and TPS in patients with PD‐L1+ CTCs ≥ 3/5 mL (*n* = 31). The results showed that the TPS was still lower than CTC PD‐L1+ percentage (median: 1% vs. 58%; *p* = 0.001), with no correlation (*r* = 0.041, *p* = 0.827; Figure S2b). Consequently, the PD‐L1 status of CTCs appears to be independent of PD‐L1 expression in primary tumor tissue.

Considering that the tumor tissue obtained by FNB may underestimate the expression of PD‐L1 in tumor tissue, we further divided the patients into two groups: TPS ≥ 50% and TPS < 50%. Results revealed that in patients with TPS < 50% (*n* = 35), TPS was significantly lower than the percentage of PD‐L1+ CTCs (median 0% vs. 56%, *p* < 0.001; Figure S1b), with no significant correlation (*r* = 0.023, *p* = 0.885; Figure S2c). Conversely, in patients with TPS ≥ 50% (*n* = 7), TPS was significantly higher than the percentage of PD‐L1+ CTCs (median 85% vs. 38%, *p* = 0.016) (Figure S1c), and similarly, no significant correlation was observed (*r* = 0.152, *p* = 0.745) (Figure S2d).

### 3.3 Correlation of CTC Subtypes With PD‐L1 Expression

**FIGURE 2:**
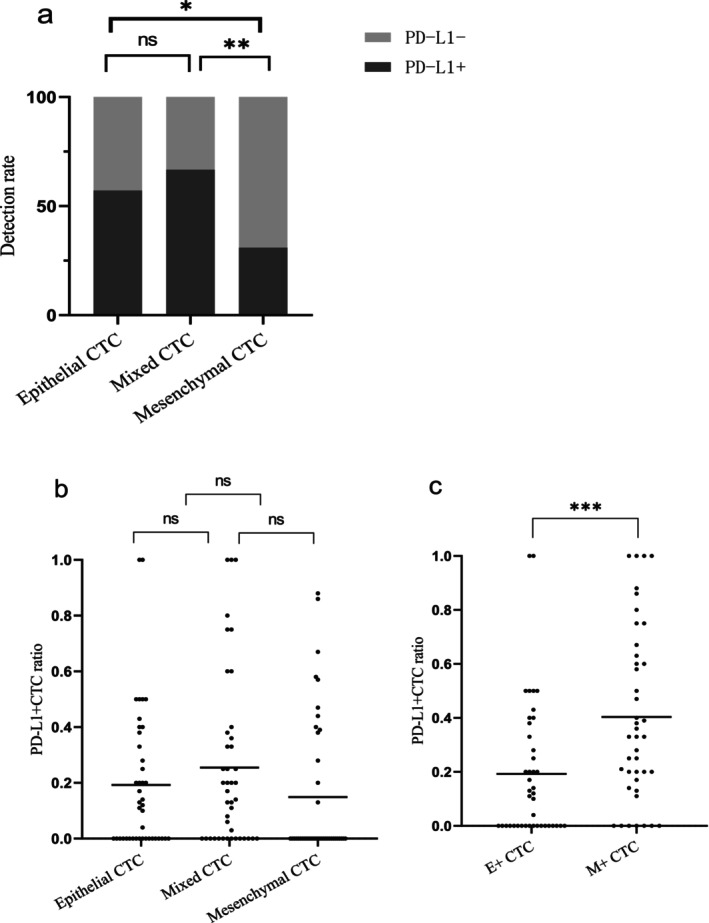
Differences in the detection rate and PD‐L1+ CTCs ratio among different CTC subtypes. (a) There was no significant difference in the detection rate of PD‐L1+ CTCs between epithelial and mixed CTCs, but both were significantly higher than the detection rate of PD‐L1+ mesenchymal CTCs. (b) There were no statistically significant differences among epithelial, mixed, and mesenchymal CTCs in the PD‐L1+ CTC ratio. (c) M+ CTCs exhibited higher PD‐L1+ CTCs proportion than E+ (33.3% vs 11.3%; *p* = 0.001). *p < 0.05, **p < 0.01, ***p < 0.001.

The detection rates of PD‐L1 expression in three distinct CTC subtypes were compared. The results demonstrated that the PD‐L1 detection rates differed significantly among CTC subtypes: epithelial 57%, mixed 67%, mesenchymal 31% (χ^2^ = 11.5, *p* = 0.003; Figure 2a). Pairwise comparisons (Bonferroni‐adjusted) revealed there was no statistically significant difference in the detection rates of PD‐L1‐positive CTCs between epithelial and mixed CTCs (χ^2^ = 0.81, *p* = 0.369). However, both epithelial and mixed CTCs exhibited higher detection rates compared to mesenchymal CTCs (χ^2^ = 5.85, *p* = 0.016 vs. χ^2^ = 10.72, *p* = 0.001; Figure 2a). A comparison was made between the proportion of PD‐L1‐positive CTCs and the total number of CTCs in the three subtypes. Non‐parametric tests revealed no statistical difference among the three groups (*F* = 1.62, *p* = 0.203; Figure 2b). Considering that the detection rate of mesenchymal CTCs is inherently low, which may lead to statistical bias, and that mixed CTCs also exhibit certain mesenchymal characteristics, we grouped mixed and mesenchymal CTCs as M+ phenotype and epithelial CTCs as E+ phenotype. M+ CTCs exhibited a higher PD‐L1+ CTCs proportion than E+ (33.3% vs. 11.3%; *p* = 0.001; Figure 2c). Notably, mesenchymal CTCs strongly correlated with PD‐L1 expression (*r* = 0.59, *p* < 0.001), exceeding mixed CTC correlation (r = 0.35, *p* = 0.024; Figure S3).

### 3.4 Impact of Tumor Invasiveness/Metastatic Potential and Primary Tumor Site on PD‐L1 Expression Discordance Between Tumor Tissue and CTC

To quantify the discrepancy in PD‐L1 expression between tumor tissue and CTC, we introduced a “PD‐L1 discordance score”, defined as the absolute value of the difference between the percentage of PD‐L1+ CTC (CTC PD‐L1%) and the TPS. Tumor burden metrics (MTV, WMTV, TLG, WTLG) and histological subtypes (adenocarcinoma vs. squamous cell carcinoma) were used as indirect surrogates for tumor invasiveness/metastatic potential.

Spearman's rank correlation analysis revealed that the correlation coefficients (r) between FDG tumor burden parameters (MTV, WMTV, TLG, WTLG) and the PD‐L1 discordance score were 0.084, −0.253, 0.091, and −0.295, with corresponding *p*‐values of 0.597, 0.106, 0.566, and 0.650, respectively. After excluding patients with extremely low CTC counts (< 3 CTCs/5 mL), the remaining 31 patients were subjected to further Spearman's rank correlation analysis. The results showed that the correlation coefficients (r) between tumor burden parameters (MTV, WMTV, TLG, WTLG) and the discordance score were 0.219, −0.152, 0.265, and −0.226, with *p*‐values of 0.237, 0.414, 0.150, and 0.222, respectively.

Regarding metabolic activity parameters, Spearman's rank correlation analysis demonstrated that the correlation coefficients (*r*) of SUVmax, SUL, and SUVmean with the discordance score were 0.128, 0.103, and 0.072, with *p*‐values of 0.418, 0.515, and 0.649, respectively. In the sensitivity analysis excluding patients with < 3 CTCs/5 mL (*n* = 31), the correlation coefficients (*r*) of SUVmax, SUL, and SUVmean with the discordance score were 0.208, 0.210, and 0.304, with *p*‐values of 0.262, 0.200, and 0.097, respectively.

The median PD‐L1 discordance scores were 61% in the adenocarcinoma group and 47% in the squamous cell carcinoma group. The Mann–Whitney U test showed no significant difference between the two groups (*Z* = −0.738, *p* = 0.461). This result remained consistent after excluding patients with < 3 CTCs/5 mL (*n* = 31), with *Z* = −0.641 and *p* = 0.521.

Due to the lack of metastatic tissue samples (only primary lung tumor tissues were collected in this study), the direct impact of lesion site on PD‐L1 discordance could not be evaluated. Therefore, we analyzed the primary tumor location (left lung vs. right lung) of all patients and calculated the corresponding PD‐L1 discordance scores. The median discordance scores were 50% for left lung primaries and 62% for right lung primaries, with no statistically significant difference (*Z* = −0.518, *p* = 0.537).

### 3.5 Correlation Between FDG Metabolic Parameters and PD‐L1 Expression in Tumor Tissue

All primary lesions showed FDG avidity. Metabolic parameters are summarized in Table 1. The correlation between PD‐L1 expression in primary tumor tissue and PET/CT metabolic parameters is shown in Table S1. The TPS positively correlated with SUVmax (*r* = 0.684, *p* < 0.001) and SUL (*r* = 0.603, *p* = 0.002), but not with tumor burden metrics (all *p* > 0.05).

### 3.6 Differences in CTC and FDG Metabolic Parameters in Patients With Different TPS Levels in Tumor Tissue

**FIGURE 3:**
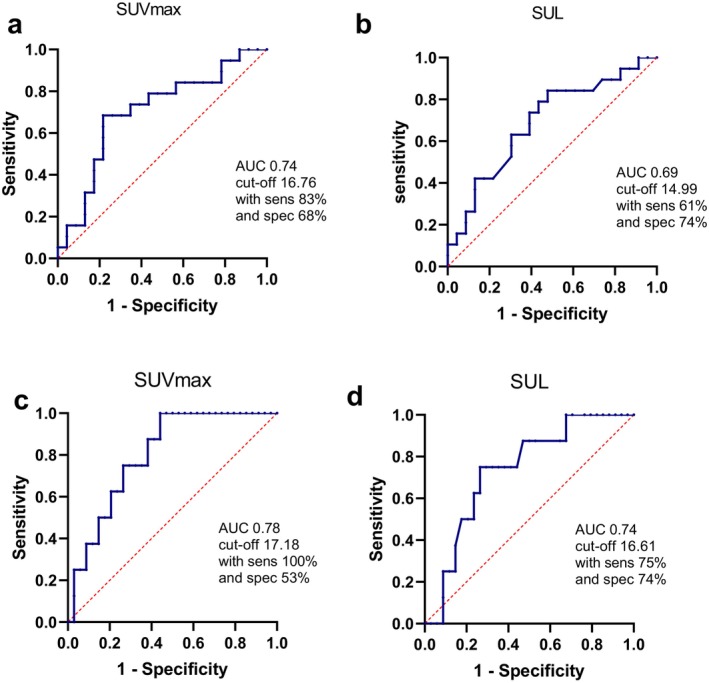
ROC curves for SUVmax, SUL in predicting tumor tissue PD‐L1 expression based on primary tumor in NSCLC patients. (a, b) At a TPS threshold of 1%, the area under the curve (AUC) for SUVmax and SUL was 0.736 and 0.690, respectively. The corresponding cut‐off values at the maximum Youden index were 16.76 (sensitivity: 83%, specificity: 68%) and 14.99 (sensitivity: 61%, specificity: 74%), respectively. (c, d) At a TPS threshold of 50%, the AUC for SUVmax and SUL was 0.783 and 0.735, respectively. The corresponding cut‐off values were 17.18 (sensitivity: 100%, specificity: 53%) and 16.61 (sensitivity: 75%, specificity: 74%), respectively.

Patients were stratified by primary tumor PD‐L1 TPS: < 1% (*n* = 14.33%), 1%–49% (*n* = 21.50%), ≥ 50% (*n* = 7.17%). Table S2 shows the mean and standard error or median of tumor metabolic parameters and PD‐L1 expression in CTCs. Results showed that SUVmax increased with TPS (15.84 vs. 18.63 vs. 26.84; *p* < 0.001), with differences between < 1% and ≥ 50% groups (*p* = 0.005). SUL showed similar trends (12.35 vs. 14.13 vs. 19.99; *p* < 0.001), differing only between < 1% and ≥ 50% (*p* = 0.022). There were no statistically significant differences between TPS and metabolic parameters reflecting tumor burden (MTV, WMTV, TLG, WTLG) and PD‐L1 expression in CTCs (*p* > 0.05). Using a threshold of 1%, patients were divided into TPS ≥ 1% (*n* = 21.50%) and TPS < 1% (*n* = 21.50%) groups (Table S3). TPS ≥ 1% group had higher SUVmax (21.91 vs. 16.33; *p* = 0.012) and SUL (16.36 vs. 12.83; *p* = 0.037) than TPS < 1% group. There were no differences in tumor burden (MTV/TLG/WMTV/WTLG) or PD‐L1 expression in CTCs (all *p* > 0.05). To determine the sensitivity and specificity of FDG metabolic parameters in predicting PD‐L1 expression at the 1% threshold, ROC curves were constructed. The AUC of SUVmax and SUL was 0.736 (cutoff 16.76; sensitivity 83%, specificity 68%) and 0.690 (cutoff 14.99; sensitivity 61%, specificity 74%) respectively (Figure 3a,b). Univariate logistic regression analysis showed that SUVmax and SUL were significant independent predictors of primary tumor PD‐L1 expression (*p* = 0.021 and 0.045, respectively). At 50% TPS threshold, SUVmax was higher in TPS ≥ 50% group (*n* = 7.17%) than in TPS < 50% group (*n* = 35.83%) (24.91 ± 5.93 vs. 18.09 ± 7.11; *p* = 0.016), but there was no significant difference in SUL (Table S4). Univariate logistic regression analysis showed that SUVmax was a significant independent predictor of TPS (*p* = 0.026). The AUC of SUVmax and SUL was 0.783 (cutoff 17.18; sensitivity 100%, specificity 53%) and 0.735 (cutoff 16.61; sensitivity 75%, specificity 74%) respectively (Figure 3c,d).

### 3.7 Value of FDG Metabolic Parameters, CTCs and Its Subtypes in Predicting Early Response to NSCLC Immunotherapy

After 8 weeks of ICI treatment in 42 NSCLC patients, 20 (48%) exhibited an early tumor response and 22 (52%) did not. Clinical data, FDG metabolic parameters, and CTC characteristics were compared between the two groups (Table 2). Statistical results showed that responders had higher baseline SUVmax (*p* < 0.05) and SUL (*p* < 0.05), but lower PD‐L1+ mixed CTCs (*p* < 0.05) (Figure S4). No significant differences were found in tissue PD‐L1 expression (TPS ≥ 50%), demographics, other FDG parameters (MTV, TLG, WMTV, WTLG), other CTC subtypes, or treatment regimen (*p* > 0.05).

**TABLE 2:** Differences between patients with or without early response at 8 weeks.

Factors	Groups	Response (*N* = 20)	No response (*N* = 22)	*p*
Age		67 ± 9.62	68 ± 8.11	0.504^a^
Gender	Male	13	16	0.588^b^
	Female	7	6	
ECOG score	0	8	11	0.501^b^
	1	11	11	
	2	1	0	
Smoking	Yes	13	14	0.927^b^
	No	7	8	
Diameter max	—	4.99 ± 2.18	5.87 ± 2.60	0.245^a^
TNM	III	9	15	0.129^b^
	IV	11	7	
Histology	AC	6	12	0.108^b^
	SCC	14	10	
PD‐L1 TPS	≥ 50%	5	2	0.229^b^
	< 50%	15	10	
Total CTCs/5 mL	—	9	9	0.752^c^
Epithelial CTCs/5 mL	—	5 (0–11)	3 (0–15)	0.089^c^
Mixed CTCs/5 mL	—	2 (0–7)	3 (0–10)	0.074^c^
EesenchymalCTCs/5 mL	—	0 (0–23)	0 (0–25)	0.288^c^
PD‐L1 + CTCs/5 mL	—	4 (1–25)	5 (0–18)	0.742^c^
PD‐L1 + Epithelial CTCs/5 mL	—	2 (0–8)	2 (0–4)	0.480^c^
PD‐L1 + Mixed CTCs/5 mL	—	0 (0–5)	2 (0–5)	0.008^c^
PD‐L1+ Mesenchymal CTCs/5 mL	—	0 (0–20)	0 (0–15)	0.252^c^
SUVmax	—	21.83 ± 7.34	16.38 ± 6.50	0.015^a^
SUL	—	16.64 ± 1.20	13.05 ± 1.09	0.033^a^
MTV	—	80.56 (8.56–471.26)	106.83 (17–654.59)	0.351^c^
WMTV	—	198.00 (66.87–929.26)	288.28 (30.98–3876.24)	0.435^c^
TLG	—	204.93 (20.43–4693.75)	763.73 (61.27–3685.34)	0.669^c^
WTLG	—	2331.64 (186.79–425,601.08)	1507.09 (104.65–456,451.30)	0.562^c^
Treatment	Immunotherapy combine with chemotherapy	18	19	0.716^b^
	Immunotherapy	2	3	

^a^ Independent samples *t*‐test.

^b^ Non‐parametric rank‐sum test.

^c^ Chi‐squared test.

**FIGURE 4:**
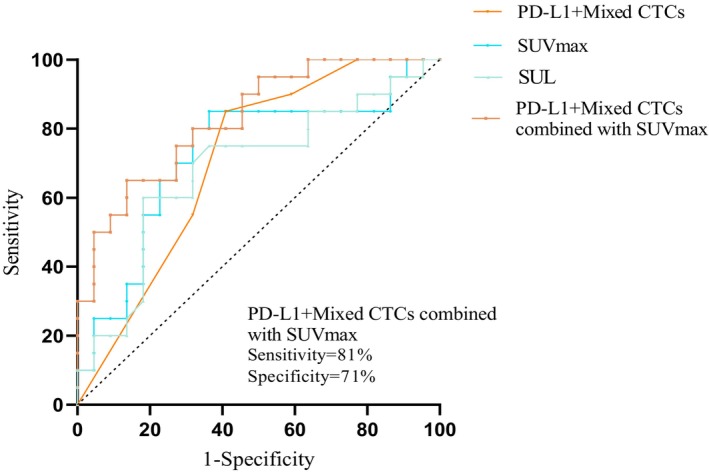
ROC curves for SUVmax, SUL, and PD‐L1+ mixed CTCs in predicting tumor early response to ICI therapy in NSCLC patients. SUVmax and PD‐L1+ mixed CTCs were combined using the highest Youden's index, and an ROC curve was plotted. The area under the corresponding curve was 0.827, with a sensitivity of 81% and a specificity of 71%.

ROC analysis for predicting early response (Figure 4) yielded AUCs of 0.725 (SUVmax; cutoff 16.45; sensitivity 86%, specificity 64%), 0.684 (SUL; cutoff 14.34; sensitivity 76%, specificity 67%), and 0.710 (PD‐L1+ mixed CTCs; cutoff 2/5 mL; sensitivity 81%, specificity 57%). The combination of SUVmax and PD‐L1+ mixed CTCs demonstrated superior predictive efficacy (AUC = 0.827; sensitivity 81%, specificity 71%). Delong test confirmed this combination significantly outperformed SUL (*p* = 0.017), but showed no significant difference versus SUVmax or PD‐L1+ mixed CTCs alone (*p* > 0.05).

### 3.8 Prognostic Value of FDG Metabolic Parameters, CTC and Its Subtypes in NSCLC Patients Treated With Immunotherapy

**FIGURE 5:**
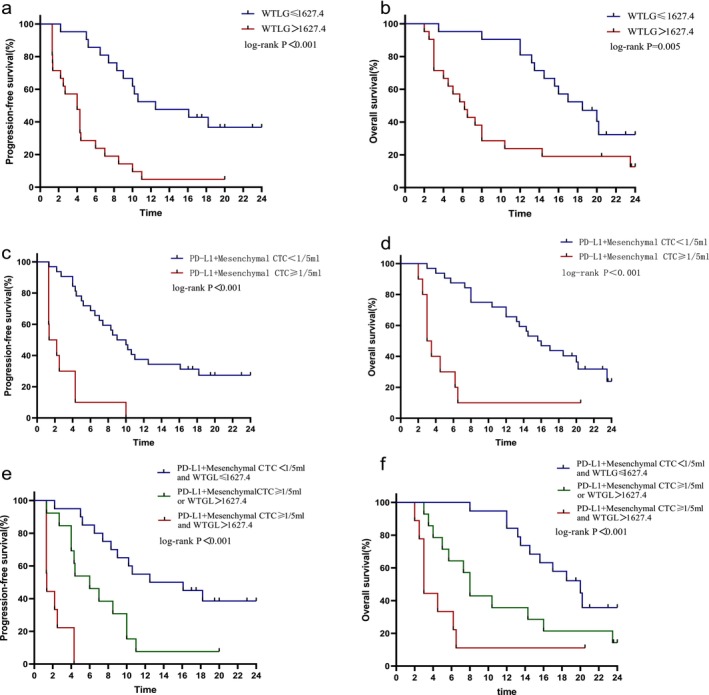
Kaplan‐Meier curves for PFS and OS in relation to WTLG (a, b), PD‐L1+ mesenchymal CTCs (c, d), and WTLG combined with PD‐L1+ mesenchymal CTCs (e, f). Log‐rank test analysis showed statistically significant differences among different groups (*p* < 0.05).

At final follow‐up, 33 (79%) NSCLC patients experienced progression and 31 (74%) died. Median PFS was 7.2 months (range: 1.3–24) and median OS was 13.4 months (range: 2–24). A Cox regression model was constructed, incorporating all quantitative PET/CT metabolic parameters and CTCs. Univariate analysis revealed that distant metastasis (HR = 2.105, 95% CI 1.116–3.423, *p* = 0.025), the total CTCs (HR = 1.052, 95% CI 1.014–1.092, *p* = 0.005), the mesenchymal CTCs (HR = 1.109, 95% CI 1.056–1.165, *p* < 0.001), and the PD‐L1+ mesenchymal CTCs (HR = 5.796, 95% CI 2.551–13.222, *p* < 0.001), WMTV (HR = 2.434, 95% CI 1.206–4.912, *p* = 0.013), and WTLG (HR = 4.362, 95% CI 2.090–9.106, *p* < 0.001) had significant effects on PFS. Multivariate analysis identified PD‐L1+ mesenchymal CTCs (HR = 5.520, 95% CI: 1.993–15.291; *p* = 0.001) and WTLG (HR = 4.315, 95% CI: 1.864–9.991; *p* < 0.001) as independent predictors. In a similar manner, distant metastasis (HR = 2.132, 95% CI: 1.137–4.125, *p* = 0.020), the total CTCs (HR = 1.045, 95% CI 1.008–1.083, *p* = 0.015), the mesenchymal CTCs (HR = 1.080, 95% CI 1.033–1.128, *p* < 0.001), the PD‐L1+ mesenchymal CTCs (HR = 4.491, 95% CI 1.973–10.222, *p* < 0.001), WMTV (HR = 2.548, 95% CI 1.230–5.279, *p* = 0.012) and WTLG (HR = 2.719, 95% CI 1.303–5.673, *p* = 0.008)were factors for OS, while only PD‐L1+ mesenchymal CTCs independently predicted OS (HR = 2.880, 95% CI: 1.124–7.381; *p* = 0.028) (Table 3). However, the 95% confidence intervals for these independent predictors were relatively wide, particularly for PD‐L1+ mesenchymal CTCs. This width reflects uncertainty in estimating the true effect size given the current sample limitations, potentially affecting result robustness. To evaluate the stability and variability of key covariate effect estimates in the multivariable Cox model, we performed bootstrap resampling (*B* = 1000 iterations) with calibration curve (Figure S5) and ROC analyses (Figure S6). For 12‐month PFS prediction, PD‐L1+ mesenchymal CTCs and WTLG demonstrated strong calibration (apparent AUC = 0.822), showing close agreement between predicted and observed outcomes without systematic risk overestimation or underestimation. Bootstrap validation yielded a mean AUC of 0.821 (95% CI: 0.714–0.917), confirming stable predictive performance and maintained high discrimination post‐validation (Figure S6a). Similarly, for 12‐month OS prediction, PD‐L1+ mesenchymal CTCs showed strong calibration (apparent AUC = 0.893) with well‐aligned predictions and no systematic bias. The bootstrap‐validated mean AUC was 0.880 (95% CI: 0.655–0.975), indicating reliable performance preservation and robust discriminative ability after internal validation (Figure S6b). Using median cut‐offs (WTLG: 1627.4 g; PD‐L1+ mesenchymal CTCs: 1/5 mL), Kaplan–Meier analysis revealed significantly longer median PFS and OS in patients with low vs. high biomarker levels: WTLG‐low: PFS 12.5 months, OS 18.5 months vs. WTLG‐high: PFS 4.0 months, OS 6.2 months (*p* < 0.05; Figure 5a,b); CTC‐low: PFS 9.5 months, OS 15.8 months vs. CTC‐high: PFS 1.8 months, OS 3.3 months (*p* < 0.05; Figure 5c,d). Combining biomarkers stratified patients into three prognostic groups (Table 4): Low‐risk (WTLG‐low and CTC‐low; *n* = 20, 48%): PFS 12.5 months, OS 18.5 months; High‐risk (WTLG‐high and CTC‐high; *n* = 13, 31%): PFS 1.35 months, OS 3.0 months; Intermediate‐risk (elevated either marker; *n* = 9, 21%): PFS 6.0 months, OS 8.0 months. Log‐rank testing confirmed significant survival differences among groups (*p* < 0.05; Figure 5e,f). The PET/CT and CTC images of typical patients are shown in Figures S9–S11. To evaluate the stability and variability of key covariate effect estimates in the multivariable risk‐stratification model, we performed bootstrap resampling (*B* = 1000 iterations) with calibration curve (Figure S7) and ROC analyses (Figure S8). For 12‐month PFS prediction, the combination of PD‐L1+ mesenchymal CTCs and WTLG demonstrated strong calibration (apparent AUC = 0.821), showing good agreement between predicted and observed outcomes without systematic overestimation or underestimation of risk. Bootstrap validation yielded a mean AUC of 0.823 (95% CI: 0.716–0.914), indicating stable predictive performance and preserved discriminative ability post‐validation (Figure S8a). Similarly, for 12‐month OS prediction, the combined biomarkers showed strong calibration (apparent AUC = 0.875) with well‐aligned predictions and no systematic bias. The bootstrap‐validated mean AUC was 0.874 (95% CI: 0.751–0.966), confirming robust predictive performance and maintained high discrimination following internal validation (Figure S8b).

**TABLE 3:** Uni‐ and multivariate analyses for progression‐free and overall survival in all patients.

	Univariate		Multivaiate		Univariate		Multivaiate	
	HR (95% CI)	*p*	HR (95% CI)	*p*	HR (95% CI)	*p*	HR (95% CI)	*p*
	Progession‐free survival				Overall survival			
Total CTCs/5 mL (> 9 vs. ≤ 9)	1.052 (1.014–1.092)	0.005	—	—	1.045 (1.008–1.083)	0.015	—	—
Epithelial CTCs/5 ml (< 4 vs. ≥ 4)	1.048 (0.949–1.157)	0.77	—	—	1.028 (0.926–1.141)	0.606	—	—
Mixed CTCs/5 mL (< 3 vs. ≥ 3)	0.925 (0.824–1.037)	0.179	—	—	0.967 (0.864–1.082)	0.557	—	—
Mesenchymal CTCs/5 mL (< 1 vs. ≥ 1)	1.109 (1.056–1.165)	< 0.001	—	—	1.080 (1.033–1.128)	< 0.001	—	—
PD‐L1 + CTCs/5 mL (> 5 vs. ≤ 5)	1.683 (0.823–3.442)	0.154	—	—	1.622 (0.785–3.355)	0.192	—	—
PD‐L1+ epithelial CTCs/5 mL (> 2 vs. ≤ 2)	1.016 (0.499–2.069)	0.964	—	—	0.914 (0.437–1.914)	0.812	—	—
PD‐L1+ mixed CTCs/5 mL (> 1 vs. ≤ 1)	0.490 (0.232–1.035)	0.062	—	—	0.711 (0.343–1.474)	0.359	—	—
PD‐L1+ mesenchymal CTCs/5 mL (> 1 vs. ≤ 1)	5.796 (2.541–13.222)	< 0.001	5.520 (1.993–15.291)	0.001	4.491 (1.973–10.222)	< 0.001	2.880 (1.124–7.381)	0.028
SUVmax (> 17.9 vs. ≤ 17.9)	1.203 (0.599–2.416)	0.604	—	—	1.150 (0.565–2.340)	0.699	—	—
SUL (> 14.3 vs. ≤ 14.3)	1.163 (0.584–2.318)	0.667	—	—	1.105 (0.543–2.246)	0.783	—	—
SUVmean (> 5.6 vs. ≤ 5.6)	2.065 (0.90–3.275)	0.061	—	—	2.279 (0.085–3.585)	0.105	—	—
MTV (> 97.8 vs. ≤ 97.8)	0.830 (0.411–1.675)	0.603	—	—	1.203 (0.592–2.445)	0.610	—	—
WMTV (> 253.4 vs. ≤ 253.4)	2.434 (1.206–4.912)	0.013	—	—	2.548 (1.230–5.279)	0.012	—	—
TLG (> 643.3 vs. ≤ 643.3)	1.264 (0.637–2.506)	0.503	—	—	1.219 (0.599–2.477)	0.585	—	—
WTLG (> 1627.4 vs. ≤ 1627.4)	4.362 (2.090–9.106)	< 0.001	4.315 (1.864–9.991)	< 0.001	2.719 (1.303–5.673)	0.008	—	—
Treatment (immunotherapy combine with chemotherapy vs. Immunotherapy)	1.216 (0.434–3.420)	0.712	—	—	1.152 (0.392–3.381)	0.805	—	—
Distant metastasis (Yes vs. No)	2.105 (1.116–3.423)	0.025	—	—	2.132 (1.137–4.125)	0.020	—	—

*Note:* The *p*‐value indicates the statistical significance of the hazard ratio.

Abbreviations: CI = confidence interval, HR = hazard ratio.

**TABLE 4:** The predictive value of WTLG combined with the PD‐L1+ mesenchymal CTCs for progression‐free survival and overall survival.

	*N*	PFS				OS			
		Median	HR	95% CI	*p*	Median	HR	95% CI	*p*
PD‐L1+ mesenchymal CTCs < 1/5 mL and WTGL < 1627.4	20	12.5	0.347	0.165–0.728	< 0.001	18.5	0.325	0.154–0.686	< 0.001
PD‐L1+ mesenchymal CTCs ≥ 1/5 mL or WTGL ≥ 1627.4	13	6.0	1.618	0.785–3.337		8.0		5.364–10.636	
PD‐L1+ mesenchymal CTCs ≥ 1/5 mL and WTGL ≥ 1627.4	9	1.35	9.181	3.499–27.951		3.0	3.955	2.513–3.487	

*Note:* The *p*‐value indicates the statistical significance of the hazard ratio.

Abbreviations: CI = confidence interval, HR = hazard ratio, OS = overall survival, PFS = progression‐free survival.

## 4 Discussion

This study focuses on the urgent need for efficacy and prognosis prediction in patients with intermediate and advanced NSCLC in the era of immunotherapy and explores the value of CTCs and FDG metabolic parameters during ICI‐based treatment. We observed that PD‐L1 expression in CTCs and primary tumor tissue lacked significant correlation, and that mesenchymal‐biased CTC phenotypes exhibited higher PD‐L1 expression. However, the discordance between tissue PD‐L1 and CTC PD‐L1 should be interpreted cautiously because the two sample types were evaluated using different detection principles. Thus, the observed discrepancy may reflect both biological heterogeneity and methodological variation. The PD‐L1 + mixed CTCs were the most responsive CTC phenotypes in which cells evade immune surveillance. EMT‐PD‐L1^+^ CTC combinations may identify poor early responders. SUVmax/SUL positively correlates with tumor tissue PD‐L1 (predicting TPS with high sensitivity but moderate specificity), though FDG uptake reflects multifactorial influences. While combining SUVmax/SUL with PD‐L1+ mixed CTCs showed no overall predictive gain, their complementary perspectives suggest utility in multimodal models. Furthermore, PD‐L1+ mesenchymal CTCs outperformed FDG parameters as independent predictors of both PFS and OS. It may therefore be a stronger predictor of disease progression and risk of death as a more central factor. Critically, pretreatment PD‐L1+ mesenchymal CTCs combined with WTLG enabled significant prognostic stratification, establishing their clinical value for risk assessment.

### 4.1 Correlation Between CTC and PD‐L1 Expression in Tumor Tissue of Matched Patients

Using multi‐probe CTC detection, we achieved a 98% PD‐L1+ CTC detection rate, which was higher than EpCAM‐based platforms (30%–60%) [23, 43, 44] and primary tumor PD‐L1 positivity [22, 23, 45]. Besides, the proportion of PD‐L1+ CTCs was also significantly higher than the PD‐L1 expression proportion in primary tumor tissues, which aligns with findings from previous studies using similar detection techniques [46, 47]. Moreover, our study found no significant association between PD‐L1 expression in primary tumor tissue and CTCs, a result consistent with conclusions from previous studies [22, 23, 45]. Nevertheless, this result should not be interpreted as evidence of biological divergence alone, because methodological differences between tissue IHC and CTC RNA‐ISH may also contribute to the lack of concordance. To explore the potential contributors to this finding, we discussed both biological and technical factors as follows.

#### 4.1.1 Sampling Bias From FNB

We fully acknowledge that FNB‐derived samples may not fully capture tumor heterogeneity [48]. To further investigate this issue, patients were stratified into two groups based on TPS cutoff of 50%. Results showed that in tumors with inherently low PD‐L1 expression (TPS < 50%), FNB sampling bias toward primary sites likely underestimates systemic immune evasion potential, whereas CTCs better integrate metastatic heterogeneity with frequent PD‐L1 upregulation. Conversely, in high‐TPS tumors (≥ 50%), PD‐L1 + CTC proportions may be suppressed due to enhanced immune‐mediated clearance of PD‐L1+ CTCs or lower CTC shedding rates. Consequently, the clinical significance of PD‐L1 expression in tumor cells may be distinct between tumor tissue and CTCs. These differences may partly reflect sampling bias from limited primary tumor biopsy material and the possibility that CTCs represent disseminated or metastatic tumor‐cell populations. However, because tissue PD‐L1 and CTC PD‐L1 were assessed using different methodologies, these findings cannot by themselves establish that the observed discordance is caused by biological heterogeneity alone. Instead, CTC PD‐L1 assessment should be considered a potentially complementary approach for characterizing systemic tumor immune features, particularly when primary biopsy tissue is limited or metastatic tissue is unavailable. When the TPS of the patient is low (< 50%), we could supplement the analysis of the systemic immune landscape with the expression of PD‐L1 in CTCs. However, the results of Ilie et al. [44] differ from our study, indicating good concordance between PD‐L1 expression in CTCs and matched tumor tissue. It is important to note that the study used the SP142 antibody for PD‐L1 detection in tumor tissues, an antibody with lower sensitivity for detecting PD‐L1 expression within tumor cells. PD‐L1 expression ≥ 1% was only observed in 10 out of 71 patients (14%), a rate far lower than the expected 50%–60% reported in the literature [17].

#### 4.1.2 The Impact of the EMT Process on PD‐L1 Expression in CTCs

Metastasizing tumor cells leverage EMT to enhance plasticity, survival in circulation, and immune evasion [49, 50]. We investigated the link between EMT phenotypes in CTCs and PD‐L1 expression. Analysis of CTC subtypes from patient samples revealed: While PD‐L1+ CTCs were detected across all phenotypes, regrouping analysis showed PD‐L1+ proportion was significantly higher in M+ CTCs (mixed+mesenchymal, 33.3%) than E+ CTCs (11.3%) (*p* < 0.05). Besides, the positive correlation between EMT degree and PD‐L1 expression was strongest in mesenchymal CTCs (*r* = *r* = 0.59, *p* < 0.001). Consequently, we hypothesize that there is an intrinsic link between EMT in CTCs and PD‐L1 expression, which, in turn, indirectly reveals that PD‐L1 expression in CTCs gradually increases with changes in the EMT process. This hypothesis is further substantiated by the findings of previous studies at the molecular level. Liu et al. [20] were the first to identify the co‐expression of mesenchymal CTCs and PD‐L1 in gastric tumor cells. The results demonstrated that the PD‐L1+ expression level of mesenchymal CTCs was significantly higher than that of epithelial CTCs. The study observed a significant decrease in the expression of mesenchymal phenotype markers in tumor cells following the PD‐L1 gene knockout. Additionally, a strong correlation was identified between PD‐L1 mRNA expression and the status of EMT, along with the migration and invasion capabilities of tumor cells. Raimondi et al. [51] studied CTCs from 15 patients with metastatic NSCLC and found a significant increase in the proportion of PD‐L1+ expression in CTCs undergoing EMT. PD‐L1+ CTCs displayed distinct spindle morphology (morphological evidence of EMT), suggesting bidirectional EMT‐PD‐L1 crosstalk. Similarly, Polioudaki et al. [52] reported a higher PD‐L1+ frequency in mesenchymal CTCs in breast cancer patients. Collectively, these studies confirm that mesenchymal‐biased phenotype CTCs (mixed/mesenchymal phenotypes) preferentially express PD‐L1, enabling immune evasion during hematogenous dissemination.

#### 4.1.3 Potential Methodological Contribution to Discordance Between Tissue IHC and CTC RNA‐ISH

An important methodological consideration is that PD‐L1 expression in primary tumor tissues and CTCs was assessed using different detection principles. In FFPE tumor tissues, PD‐L1 was evaluated by IHC using the 22C3 antibody, and TPS was calculated based on membranous protein staining in viable tumor cells. In contrast, PD‐L1 expression in CTCs was evaluated by RNA‐ISH, in which intracellular PD‐L1 mRNA signal dots were counted as positive events. Therefore, direct numerical comparison between tissue TPS and CTC PD‐L1 percentage should be interpreted with caution. The observed lack of correlation between tissue PD‐L1 and CTC PD‐L1 (*r* = 0.041, *p* = 0.827 in patients with ≥ 3 PD‐L1+ CTCs/5 mL) may reflect a combination of spatial/temporal tumor heterogeneity, differences between primary and disseminated tumor cell populations, and technical variation introduced by protein‐based versus mRNA‐based assays. Accordingly, we revised the interpretation of our findings and avoided attributing PD‐L1 discordance solely to inherent biological divergence between primary tumors and CTCs.

#### 4.1.4 The Impact of Tumor Invasiveness Potential and Metastatic Sites on PD‐L1 Expression

To clarify the contribution of clinicopathological factors to PD‐L1 expression heterogeneity, the PD‐L1 discrepancy score was employed in this study to quantify the magnitude of PD‐L1 expression differences between CTCs and primary tumor tissues. Subsequent analyses were performed to delineate the associations between this score and relevant clinicopathological variables.

Spearman's rank correlation analysis revealed that the correlation coefficients between PET/CT‐derived metabolic burden indices (MTV, WMTV, TLG, WTLG) and the PD‐L1 discrepancy score ranged from −0.295 to 0.091 (all *p* > 0.05). These results indicated that neither tumor volume nor global metabolic activity was significantly correlated with PD‐L1 expression discrepancy. To eliminate potential confounding effects of low CTC counts on the analysis, we further excluded patients with CTC counts < 3/5 mL (*n* = 31). Reassessing the correlations yielded identical non‐significant results (correlation coefficient range: −0.226 to 0.265, all *p* > 0.05), confirming the robustness of our initial findings. In parallel, no significant correlations were detected between metabolic activity parameters (SUVmax, SUL, SUVmean) and the PD‐L1 discrepancy score (correlation coefficient range: 0.072 to 0.128, all *p* > 0.05). A sensitivity analysis was conducted to validate these observations, and the outcomes remained consistent (correlation coefficient range: 0.200 to 0.304, all *p* > 0.05). Collectively, these data suggest that PD‐L1 expression discrepancy is not determined by disease stage or tumor volume. Instead, it reflects inherent biological divergences between primary tumor tissues and CTCs.

Given that metastatic lesion samples were not available for this study, the analysis was restricted to evaluating the impact of primary lesion location (left lung vs. right lung) on PD‐L1 discrepancy. The results showed that the mean discrepancy score was 50% in patients with left lung primary lesions and 62% in those with right lung primary lesions, with no statistically significant difference between the two groups (*Z* = −0.518, *p* = 0.537). This finding demonstrates that primary lesion location does not exert a measurable effect on PD‐L1 expression differences between primary tumors and CTCs.

It is important to acknowledge that CTCs may originate from systemic metastatic lesions rather than the primary tumor itself, and PD‐L1 expression may vary between primary and metastatic lesions [45]. However, metastatic tissue samples were not available in the present study, and the same RNA‐ISH method was not applied to matched FFPE tumor tissue. Therefore, future studies incorporating paired primary tumor tissue, metastatic tissue, and CTCs, ideally analyzed using harmonized or parallel detection platforms, will be essential to clarify the relative contributions of biological heterogeneity and methodological variation to PD‐L1 discordance.

Furthermore, we evaluated the association between histological subtype and PD‐L1 discrepancy. The mean discrepancy score was 61% in adenocarcinoma patients (*n* = 32) and 47% in squamous cell carcinoma patients (*n* = 10). The Mann–Whitney U test confirmed no statistically significant difference between the two subtypes (*Z* = −0.738, *p* = 0.461). Excluding patients with low CTC counts did not alter this non‐significant trend (*Z* = −0.641, *p* = 0.521), indicating that PD‐L1 expression discrepancy was not significantly associated with histological subtype in this cohort. This observation aligns with previous reports demonstrating comparable PD‐L1 heterogeneity among NSCLC subtypes [45, 48]. Together, these exploratory analyses suggest that the PD‐L1 discrepancy between primary tumor tissues and CTCs is unlikely to be driven solely by the examined clinical variables, but the contribution of methodological differences remains an important limitation.

In summary, the examined clinicopathological variables did not significantly explain the PD‐L1 discrepancy between primary tumor tissues and CTCs. This finding supports the need to consider additional factors, including spatial and temporal tumor heterogeneity, CTC origin, EMT‐associated immune escape, and assay‐related differences between tissue IHC and CTC RNA‐ISH.

### 4.2 Predictive Value of FDG Metabolic Parameters and CTCs for Primary Tumor PD‐L1 Status and Early Response to ICI Therapy

This study investigated the correlation between PD‐L1 expression (using TPS thresholds of 1% and 50% [53]) and FDG PET/CT metabolic parameters in primary tumors of advanced NSCLC patients. We observed significant positive associations between rising TPS levels and increased SUVmax/SUL values (*p* < 0.05). For predicting TPS ≥ 1% positivity, SUVmax > 16.76 demonstrated high sensitivity (83%) but moderate specificity (68%), while SUL > 14.99 showed balanced moderate performance (61% sensitivity, 74% specificity). Notably, only SUVmax > 17.18 retained predictive capacity for TPS ≥ 50% positivity, achieving 100% sensitivity at the cost of reduced specificity (53%). These results underscore the inherent limitations of FDG parameters as standalone predictors due to multifactorial influences on glucose metabolism beyond PD‐L1 expression. Consequently, while metabolic markers exhibit correlative value, their clinical utility likely resides in augmenting multimodal predictive models for immunotherapy stratification rather than independent application.

Consistent with prior research [5, 7, 54], our study found that early tumor responders exhibited significantly higher SUVmax and SUL than non‐responders. Despite a degree of heterogeneity in patient and histological characteristics within and between studies, SUVmax demonstrated the strongest and most consistent association with primary tumor PD‐L1 expression and early treatment efficacy. Grizzi et al. [5] reported most rapid progressors (8/9) had baseline SUVmax ≤ 17.1 and SUVmean ≤ 8.3, with these cutoffs showing high sensitivity (88.9%–100%) but low specificity (33.3%–38.9%) for progressive disease. Similarly, Takada et al. [54] observed significantly higher response rates in NSCLC patients with SUVmax ≥ 11.1, while Evangelista et al. [7] confirmed SUVmax had the strongest association with PD among FDG parameters. In traditional therapies such as chemotherapy and radiotherapy, high baseline SUVmax usually indicates more aggressive tumor biological behavior. This means that tumor cells are highly proliferative, have abundant angiogenesis, and vigorous glycolytic metabolism. Such tumors often possess stronger metastatic potential and intrinsic resistance to cytotoxic drugs [55]. The core reason is that traditional therapies mainly target rapidly dividing tumor cells. Tumors with high metabolic activity are not only insensitive to chemotherapeutic drugs but also may proliferate and recur rapidly after treatment. Therefore, high SUVmax serves as a marker of poor prognosis. Unlike the mode of action of traditional therapies that directly kill tumor cells, the core of ICI therapy is to reactivate the host's immune system, enabling it to recognize and eliminate tumor cells. In our study, high baseline SUVmax does not merely reflect tumor aggressiveness but is more likely to indicate that the TME is in an immune‐active state, which provides favorable conditions for the efficacy of ICI therapy: high baseline FDG uptake may suggest a metabolically active and immune‐infiltrated “hot” tumor microenvironment, which may be more sensitive to immune reactivation [56, 57]. High SUVmax may stem from the metabolic demands of a large number of infiltrating immune cells (such as T cells, macrophages, etc.) in tumor tissues. Activated immune cells acquire energy through enhanced glycolytic metabolism to support their proliferation, activation, and cytokine secretion [48, 49]. In this study, patients with high SUVmax had significantly higher PD‐L1 expression levels in tumor tissues (*r* = 0.684, *p* < 0.001). High PD‐L1 expression usually implies active interactions between tumor cells and immune cells, and such “immune‐hot tumors” have a higher response rate to ICI therapy. In addition, the early “flare‐up” of inflammation after ICI initiation, which represents immune activation rather than true progression, may also lead to increased FDG uptake, which may be misinterpreted in some studies [58]. Our analysis focused on evaluating the association between baseline SUVmax (pre‐treatment) and early response (8 weeks), thus it is more likely to reflect the pre‐existing immune‐active state before treatment rather than the inflammatory response after treatment. This mechanism is consistent with the studies by Takada et al. [54] and Grizzi et al. [5], both confirming that high baseline metabolism is associated with benefits from ICI therapy. In addition to differences in treatment modes, the following factors may also lead to inconsistencies between the results of this study and some previous studies: Heterogeneity in study populations and treatment regimens: 88% of patients in this study received ICI combined with chemotherapy, while most previous studies reporting “high SUVmax is associated with poor prognosis” were based on populations receiving ICI monotherapy [11, 13]. Chemotherapeutic drugs may alter the immune state of the tumor microenvironment by inducing immunogenic cell death and upregulating PD‐L1 expression on tumor cells, thereby affecting the association between SUVmax and treatment response. In summary, SUVmax essentially reflects the level of tissue glucose uptake, and its value is influenced by various factors such as tumor cell metabolism, immune cell infiltration, and inflammatory responses [9, 54]. In ICI therapy, “immune‐related high metabolism” caused by immune cell infiltration may mask the adverse effects of the tumor cells' own high metabolism, resulting in high SUVmax being ultimately associated with favorable treatment response rather than poor prognosis. Although patients with high SUVmax have a higher early response rate, their long‐term prognosis may be affected by various factors such as tumor heterogeneity and immune resistance, which may also lead to differences in conclusions among different studies.

Conversely, tumor burden parameters (MTV, WMTV, TLG, WTLG) showed no predictive value for early tumor response, aligning with previous studies [10, 59, 60]. MTV's dependence on lesion volume (influenced by disease duration/progression) introduces individual variability, while TLG (MTV × SUVmean) inherits these limitations and exhibits increased data dispersion. Both primarily reflect metabolic activity and volume but fail to adequately represent functional immune microenvironment status due to spatial heterogeneity, immunosuppression, and threshold dependency. Thus, SUVmax and SUL emerge as more reliable predictors of NSCLC immunotherapy response than tumor burden metrics, whose clinical predictive value remains limited.

It should be noted that ICI regimens included in our study vary widely, and real‐world clinical treatment is influenced by multiple factors, preventing strict adherence to guidelines in regimen selection. Most patients in this study received ICI combined with chemotherapy, which may impact the role of immunohistochemistry and FDG metabolic parameters in predicting efficacy. First, previous studies [61, 62] indicate that paclitaxel, etoposide, and 5‐fluorouracil can upregulate PD‐L1 expression on tumor cell surfaces. The inclusion of chemotherapeutic agents may consequently distort baseline tumor tissue PD‐L1 expression. Furthermore, research [63] demonstrates that PD‐L1 exhibits significant predictive value in ICI monotherapy cohorts (OR = 5.6), whereas its predictive capacity diminishes in combination therapy groups (OR = 1.9). Although our findings revealed no significant association between baseline tumor PD‐L1 expression and early tumor response across both treatment regimens (*p* = 0.229), this result may be influenced by chemotherapeutic agents administered to the majority of patients. Second, chemotherapeutic agents downregulate GLUT1 transporters and hexokinase‐2 (HK‐2) phosphorylation, suppressing glycolytic pathways and reducing SUVmax by 25%–40% [64]. Such artificial SUV reduction may be misinterpreted as treatment sensitivity. Additionally, chemotherapy‐induced immunogenic cell death triggers non‐neoplastic inflammation (e.g., pneumonitis/necrosis), elevating local FDG uptake that could be misclassified as tumor progression [65]. Thus, chemotherapy‐mediated metabolic alterations may compromise the predictive validity of SUV changes for treatment response. However, our study specifically evaluated baseline FDG metabolic parameters for predicting early tumor response. The predictive value of baseline SUVmax remained intact regardless of treatment regimen, maintaining clinically relevant accuracy (SUVmax AUC = 0.725 vs. SUL AUC = 0.684). This observation aligns with prior investigations and confirms the preserved utility of baseline metabolic imaging for response prediction amid therapeutic heterogeneity. In a word, these findings should thus be interpreted as reflective of contemporary first‐line practice, where combination therapy predominates, rather than as biomarkers of ICI (monotherapy) response. And when comparing these findings with ICI clinical trials from other regions, such differences must be considered and adjustments made accordingly.

Beyond FDG metabolic parameters, CTC (especially subtype‐specific PD‐L1 expression) also showed predictive relevance for early immunotherapy response. Patients exhibiting higher numbers of PD‐L1+ mixed CTCs demonstrated poorer immunotherapy responses than those with other CTC subtypes. While total mixed CTCs (PD‐L1+/−) were numerically higher in non‐early responders versus early responders, this difference lacked statistical significance (*p* = 0.074). Current evidence regarding CTCs' predictive value for ICI efficacy in NSCLC remains limited and contradictory: Guibert et al. [22] prospectively found baseline PD‐L1+ CTCs in all progressing patients (*n* = 96), suggesting ICI resistance. Conversely, Janning et al. [23] observed increasing PD‐L1+ CTCs in progressors but stable/decreasing levels in responders among 127 patients, proposing PD‐L1+ CTC dynamics as resistance biomarkers. Paradoxically, Zhou et al. [24] reported significantly higher response rates (overall response rate 36.4% vs. 19.0%; disease control rate 81.8% vs. 47.6%) in pretreatment PD‐L1+ CTC patients (*n* = 49), noting ICI benefit even in IHC PD‐L1− patients with PD‐L1+ CTCs. These discrepancies likely stem from methodological heterogeneity in CTC enrichment/detection, PD‐L1 antibodies, patient cohorts, and crucially, the absence of CTC subtype‐specific PD‐L1 analysis across studies. Biologically, elevated PD‐L1 expression enables immune evasion via PI3K/Akt pathway activation [66]. As mixed CTCs represent an EMT‐intermediate state with mesenchymal features, their high PD‐L1 expression indicates adaptive immune escape and intrinsic immunotherapy resistance. Thus, PD‐L1+ mixed CTCs reflect ICI‐resistant patients requiring tailored clinical management. Assessing PD‐L1 across CTC subtypes holds promise for prognostic evaluation and personalized immunotherapy guidance, though further genomic/epigenomic characterization of these subpopulations is warranted.

Although SUV and PD‐L1+ mixed CTCs offer some predictive value for early tumor response, their specificity remains limited. Two mechanisms may contribute: First, SUV reflects glucose uptake influenced by tumor microenvironment heterogeneity, individual variations, metabolic activity, and inflammatory responses. Second, PD‐L1+ mixed CTC's poor specificity stems from dynamic microenvironmental interactions (immune infiltration, cytokine signaling, cell‐matrix crosstalk) that alter CTC phenotypes over time [67]. This instability compromises their reliability as immunotherapy biomarkers. Furthermore, technical limitations in current CTC detection methods impede comprehensive tumor characterization. Our data indicate that combining these approaches fails to significantly enhance early‐response prediction. Future efforts should prioritize developing PET‐specific probes, optimizing CTC detection technologies, and integrating liquid biopsies with multi‐omics analyses to improve accuracy.

### 4.3 The Prognostic Value of FDG Metabolic Parameters and CTCs in NSCLC Patients Treated With ICI

Univariate analysis identified distant metastasis, total CTCs, mesenchymal CTCs, PD‐L1+ mesenchymal CTCs, WMTV, and WTLG as significant factors for both PFS and OS. However, multivariate analysis revealed PD‐L1+ mesenchymal CTCs and WTLG as independent PFS predictors, while only PD‐L1+ mesenchymal CTCs independently impacted OS—a discrepancy attributable to covariate interdependencies in univariate testing.

First, these findings confirm that distant metastasis is a well‐established poor prognostic factor for advanced NSCLC [68, 69, 70], which is also supported by the results of our univariate analysis. Nevertheless, the prognostic effect of distant metastasis was overridden by the more biologically meaningful biomarkers (PD‐L1+ mesenchymal CTCs and WTLG) included in the model. The underlying reasons may be twofold: first, WTLG quantifies the total body tumor burden (including metastatic lesions), thus replacing the qualitative description of metastatic status; second, PD‐L1+ mesenchymal CTCs reflect the EMT phenotype and immune escape ability of tumors, which represent the core biological mechanisms driving metastasis and poor prognosis, and thus have a higher prognostic weight. Therefore, compared with the qualitative indicator distant metastasis, PD‐L1+ mesenchymal CTCs and WTLG are more precise prognostic biomarkers that can be used for individualized risk stratification of patients. In contrast, distant metastasis may be incorporated as a clinical baseline characteristic for patient stratification description, but it is not required to be used as an independent prognostic factor for clinical decision‐making. Second, this study corroborates prior research on PET metabolic parameters [11, 12, 13, 60] and PD‐L1 expression of CTCs [21, 22, 23, 24, 25, 26]. As metabolic parameters that can reflect the dual information of tumor volume and metabolic activity, MTV and TLG can overcome the limitation that SUVmax only provides FDG activity in a single voxel of tumor, and can reflect the metabolic state of the whole body. Previous studies based on FDG metabolic parameters have shown that metabolic parameters can be used for patient stratification, but there are differences in different research results and TLG and MTV cutoff values, which may be due to differences in population, 18F‐FDG injection dose, PET/CT procedures, and segmentation methods. In our study, WTLG ≥ 1627.4 g represents patients with larger total tumor volume and higher metabolic activity. Patients exceeding this value may have extensive metastasis, high glycolytic activity, or treatment resistant biological behavior.

While the prognostic value of CTCs remains debated due to technical heterogeneity, PD‐L1+ CTCs generally have clinical utility. Our findings specifically establish PD‐L1+ mesenchymal CTCs as prognostic markers for PFS/OS, supporting the hypothesis that co‐expression of PD‐L1 and EMT phenotype in CTCs accelerates NSCLC progression. Notably, PD‐L1+ mesenchymal CTCs outperformed epithelial or mixed CTC subtypes in prognostic efficacy, attributed to three core characteristics: mesenchymal CTCs gain strong metastatic potential via EMT and resist anoikis [49, 51], while their PD‐L1 expression enhances immune escape by binding PD‐1 on T cells [18, 66], with EMT further upregulating PD‐L1 through PI3K/Akt/STAT3 pathways [19, 20] to form an “EMT‐PD‐L1” amplification loop, in contrast to epithelial CTCs (lacking this synergy, PD‐L1 detection rate 57%) and mixed CTCs (intermediate EMT, moderate metastatic potential, 33.3% PD‐L1 positive rate) [20, 52] that have limited prognostic value; derived from the most invasive tumor subclones [45, 47], PD‐L1+ mesenchymal CTCs directly indicate systemic metastatic progression (65.8% detection rate in metastatic NSCLC vs. 0% in benign diseases), better capturing systemic tumor aggressiveness than other subtypes; the mesenchymal phenotype links to intrinsic ICI resistance via EMT‐related pathways [18, 50], and PD‐L1 overexpression mediates adaptive resistance [22, 24], while epithelial CTCs lack this dual resistance trait and mixed CTCs only partially overlap with resistance‐related features, leading to inferior prognostic performance.

Compared to tumor‐burden PET metrics, PD‐L1+ mesenchymal CTCs may better predict mortality risk. Combining pretreatment PD‐L1+ mesenchymal CTCs and WTLG showed prognostic value, consistent with three similar existing studies. Fu et al. [40] evaluated the treatment efficacy of chemotherapy in 129 patients with stage IIIB NSCLC and found that baseline CTCs, CTCs after the 1st/4th chemotherapy cycle, and WMTV before treatment initiation were significantly correlated with PFS and OS. The combination of these two markers could enhance the prognostic evaluation of stage IIIB NSCLC. Castello et al. [42] demonstrated that elevated CTCs and metabolic parameters were prognostic factors for PFS and OS in NSCLC patients treated with ICI. Conversely, Zhang et al. [38] found that TLG and CTCs were independent predictors of PFS and OS in NSCLC patients. However, the abovementioned studies did not perform detailed subtyping of CTCs, nor did they conduct further research on the PD‐L1 phenotype of CTCs. It should be noted that the modest sample size (*n* = 42) in this study limits statistical power and model stability. Following stratification by CTCs combined with WTLG, subgroup sample sizes became substantially reduced, further diminishing statistical power for these analyses. As reflected in the wide confidence intervals, effect estimates show limited precision and potential instability. However, such wide CIs represent expected consequences of limited sampling rather than model deficiencies. To address these limitations, we implemented rigorous bootstrap internal validation (*B* = 1000 iterations) to evaluate the predictive performance of PD‐L1+ mesenchymal CTCs and WTLG. The validation results support the robustness of our predictive model. Furthermore, our methodology and conclusions align with prior research by Castello et al. [42](*n* = 35), demonstrating similar predictive signatures. The proposed model retains clinical value for prognostic stratification of NSCLC patients receiving ICI therapy. Nevertheless, while the model demonstrated stability during internal bootstrap validation, its cross‐domain applicability requires verification through multicenter cohorts. Future validation in larger, multi‐institutional populations is warranted to confirm generalizability.

Notably, in exploratory prognostic studies, the use of the median as the grouping cutoff value is a widely accepted statistical strategy in the absence of clear clinical thresholds, which can effectively avoid bias caused by the lack of recognized clinical thresholds [39, 41]. This study showed that there were significant differences in PFS and OS among the three groups (*p* < 0.05), confirming the clinical relevance of this cutoff value. Furthermore, the calibration curves from bootstrap internal validation (1000 iterations) further verified the stability and predictive accuracy of the model, with a mean AUC value of > 0.82, indicating that the model has good discriminative ability and clinical practical value.

This study has the following limitations: First, the modest sample size (*n* = 42) of this single‐center study constitutes its primary limitation. Although formal sample size calculation indicated a requirement for 77 patients, strict inclusion criteria and recruitment timelines resulted in a smaller final cohort. This reduced the statistical power of our analyses, increased the risk of overfitting in multivariable models, and may have compromised the precision and generalizability of our estimates. While we employed bootstrapping internal validation to assess model stability, these findings require external validation in larger, independent cohorts. Addressing this limitation is paramount. Therefore, securing validation in independent cohorts constitutes a primary objective of our future research. We are actively pursuing funding and collaborative opportunities to conduct a dedicated multi‐center validation study. In addition, future studies should adopt advanced statistical techniques, such as penalized regression (e.g., LASSO), to optimize the selection and validation of biomarker panels from a broader range of candidate indicators. Confirming our results in diverse settings is essential before these parameters can be considered for widespread clinical implementation. Second, PD‐L1 expression in primary tumor tissue and CTCs was evaluated using different detection methods. Tissue PD‐L1 was assessed by IHC, whereas CTC PD‐L1 was assessed by RNA‐ISH. This methodological inconsistency limits our ability to distinguish biological heterogeneity from assay‐related discordance. In this cohort, matched RNA‐ISH analysis of primary FFPE tumor samples was not performed because the available diagnostic biopsy material was limited and was prioritized for routine pathological and molecular testing. Future studies should apply harmonized or parallel detection platforms, such as RNA‐ISH and IHC in both FFPE tumor tissues and CTCs, to more directly evaluate concordance. Third, all tumor tissues in this study were obtained by FNB, potentially precluding analysis of its correlation with metabolic parameters and CTCs in metastatic sites. Future studies should incorporate surgical specimens and metastatic biopsies for a more comprehensive evaluation. Fourth, only baseline data were analyzed; longitudinal assessment of FDG metabolic parameters and CTC dynamics during treatment is needed to determine their predictive value for therapeutic response and prognosis. Finally, most patients received ICI plus chemotherapy rather than monotherapy, which may confound the predictive role of immunohistochemistry and metabolic parameters. Further validation in treatment‐naïve cohorts receiving ICI alone is warranted.

## 5 Conclusion

This study highlights distinct PD‐L1 expression patterns between primary tumor tissues and CTCs in advanced NSCLC. PD‐L1 expression in mesenchymal‐biased CTCs was associated with EMT features and survival outcomes, and PD‐L1+ mixed CTCs showed predictive relevance for early immunotherapy response. However, because tissue PD‐L1 and CTC PD‐L1 were assessed by different methodologies, the observed discordance should be interpreted cautiously and may reflect both biological heterogeneity and methodological variation. The integration of pretreatment PD‐L1+ mesenchymal CTCs with WTLG may enhance prognostic stratification, but these exploratory findings require validation in larger cohorts using harmonized detection approaches and, where possible, matched primary and metastatic tumor samples.

## Author Contributions

**Momo Sun:** conceptualization, methodology, software, investigation, formal analysis, writing – original draft. **Jie Shen:** writing – review and editing, project administration, resources, supervision, funding acquisition. **Aisikaer Aikedan:** visualization, formal analysis. **Enci Ding:** resources, supervision, project administration. **Zhongying Rui:** software, data curation, validation.

## Funding

This study was supported by Tianjin Municipal Health Commission (TJWJ2025RC008) and Tianjin Municipal Science and Technology Bureau (21JCYBJC01060).

## Consent

All authors approved the final manuscript and the submission to this journal.

## Conflicts of Interest

The authors declare no conflicts of interest.

## Supporting information


**Figure S1:** Comparative analysis of the percentage of PD‐L1 expression in tumor tissue and CTCs of matched patients with advanced NSCLC. (a) In all patients (*n* = 42), percentage of PD‐L1 expression in tumor tissue was significantly lower than the percentage of PD‐L1+ CTCs (median: 0.5% vs. 56%; *p* < 0.001); (b) In patients with TPS < 50% (*n* = 35), percentage of PD‐L1 expression in tumor tissue was significantly lower than the percentage of PD‐L1+ CTCs (median 0% vs. 56%, *p*<0.001). (c) In patients with TPS ≥ 50% (*n* = 7), TPS was significantly higher than the percentage of PD‐L1+ CTCs (median 85% vs. 38%, *p* = 0.016). **p < 0.01, ***p < 0.001.
**Figure S2:** Correlation between the percentage of PD‐L1+ in tumor tissue and PD‐L1+ CTC. (a) In patients with PD‐L1+ CTC count ≥ 1/5 mL, there is no correlation between the percentage of PD‐L1+ CTC and the percentage of PD‐L1+ in tumor tissue (*r* = 0.03, *p* = 0.87). (b) In patients with PD‐L1+ CTC count ≥ 3/5 mL, there is no correlation between the percentage of PD‐L1+ CTCs and the percentage of PD‐L1+ in tumor tissue (*r* = 0.041, *p* = 0.827). (c) In NSCLC patients with TPS < 50%, there is no correlation between the percentage of PD‐L1 in tumor tissue and the percentage of PD‐L1+ CTC (*r* = 0.023, *p* = 0.885). (d) In NSCLC patients with TPS ≥ 50%, there is no correlation between the percentage of PD‐L1 in tumor tissue and the percentage of PD‐L1+ CTC (*r* = 0.152, *p* = 0.745).
**Figure S3:** Correlation between PD‐L1 expression and different Subtypes of CTCs. Red indicates positive correlation, and blue indicates negative correlation. Among PD‐L1+ CTCs, mixed CTCs and mesenchymal CTCs showed a positive correlation with PD‐L1 expression (*r* = 0.35, *p* = 0.024 and *r* = 0.59, *p* < 0.001), with the correlation between mesenchymal CTCs and PD‐L1 expression being more significant.
**Figure S4:** Tumor early response evaluation at 8 weeks based on RECISTv1.1 in NSCLC patients receiving ICI monotherapy or combined chemotherapy: differences between the early tumor response group and the no response group. (a) Patients exhibiting early tumor response had lower baseline PD‐L1+ mixed CTCs than patients with no early tumor response. (b, c) Patients exhibiting early tumor response had higher SUVmax and SUL values than patients with no early tumor response. *p < 0.05, **p < 0.01.
**Figure S5:** Calibration curves for WTLG and PD‐L1+ mesenchymal CTCs in predicting progression‐free survival (PFS) (a), and for PD‐L1+ mesenchymal CTCs in predicting overall survival (OS) (b). The plots demonstrate the calibration performance of the models, indicating agreement between predicted probabilities and observed outcomes. Blue data points cluster closely along the 45‐degree diagonal line (ideal calibration line), indicating good calibration of the Cox regression models for PFS and OS prediction. Predicted risks align well with actual events, with no evidence of systematic overestimation or underestimation of risk (Bootstrap resampling: *B* = 1000).
**Figure S6:** ROC curves for WTLG and PD‐L1+ mesenchymal CTCs in predicting progression‐free survival (PFS) (a), and for PD‐L1+ mesenchymal CTCs in predicting overall survival (OS) (b). The original ROC curves (solid red lines) yielded AUC values of 0.822 (a) and 0.893 (b). Following 1000 bootstrap resampling validations (thin gray lines), the mean AUCs were 0.821 (95% CI: 0.714–0.917) for (a) and 0.880 (95% CI: 0.655–0.975) for (b), demonstrating stable and robust predictive performance with preserved high discriminative power after internal validation.
**Figure S7:** Calibration curves for WTLG combine with PD‐L1+ mesenchymal CTCs in predicting progression‐free survival (PFS) (a) and overall survival (OS) (b). The plots demonstrate the calibration performance of the models, indicating agreement between predicted probabilities and observed outcomes. Blue data points cluster closely along the 45‐degree diagonal line (ideal calibration line), indicating good calibration of the Cox regression models for PFS and OS prediction. Predicted risks align well with actual events, with no evidence of systematic overestimation or underestimation of risk (Bootstrap resampling: *B* = 1000).
**Figure S8:** ROC curves for WTLG combine with PD‐L1+ mesenchymal CTCs in predicting progression‐free survival (PFS) (a) and overall survival (OS) (b). The original ROC curves (solid red lines) yielded AUC values of 0.821 (a) and 0.875 (b). Following 1000 bootstrap resampling validations (thin gray lines), the mean AUCs were 0.823 (95% CI: 0.716–0.914) for (a) and 0.875 (95% CI: 0.751–0.966) for (b), demonstrating stable and robust predictive performance with preserved high discriminative power after internal validation.
**Figure S9:** 18F‐FDG PET/CT and CTC images of a patient with stage IV NSCLC. The patient was a 69‐year‐old female with adenocarcinoma, treated with chemoimmunotherapy. (a) The 18F‐FDG PET/CT MIP image before treatment shows multiple foci of abnormally increased glucose metabolism throughout the body. (b) A mass in the upper lobe of the left lung (maximum transverse diameter approximately 4.75 cm) with abnormally increased glucose metabolism (SUVmax of 8.68, SUL of 6.83, MTV of 50.52 mm3, TLG of 219.76 g). The patient also had multiple metastases in the mediastinal lymph nodes (c), liver (d), and right pubic bone (e), all of which showed abnormally increased glucose metabolism (WMTV of 243.23 mm^3^, WTLG of 46,872.85 g). In addition, 25 CTCs were detected in the patient's peripheral blood before treatment, including 8 PD‐L1+ mesenchymal CTCs (f), 7 PD‐L1‐ mesenchymal CTCs (g), and 10 PD‐L1‐ mixed CTCs (h), typical examples of which are shown in the figure. The patient's PFS was 2.0 months, and OS was 4.5 months.
**Figure S10:** 18F‐FDG PET/CT and CTC images of a patient with stage III NSCLC. The patient was a 59‐year‐old male with squamous cell carcinoma, treated with immunotherapy monotherapy. (a) Pre‐treatment 18F‐FDG PET/CT MIP image demonstrates abnormally increased glucose metabolism in the right lower lobe and mediastinum. (b, c) Images show a nodule in the right lower lobe (maximum diameter approximately 3.0 cm) with abnormally increased glucose metabolism (SUVmax 14.18, SUL 12.00, MTV 26.18 mm^3^, TLG 96.13 g). The patient also presented with mediastinal (subcarinal) lymph node metastasis (d, e), and all metastatic lesions showed abnormally increased glucose metabolism (WMTV 120.13 mm^3^, WTLG 528.07 g). A total of 8 CTCs were detected in the patient's peripheral blood prior to treatment, including 1 PD‐L1+ epithelial CTC (f), 5 PD‐L1‐ epithelial CTCs (g), and 2 PD‐L1‐ mixed‐type CTCs (h), with typical examples shown in the figure. As of the follow‐up date, the patient was alive and had not experienced progression.
**Figure S11:** 18F‐FDG PET/CT and CTC images of a patient with stage IV NSCLC. The patient was a 47‐year‐old male with adenocarcinoma, treated with immunotherapy combined with chemotherapy. (a) The MIP image of 18F‐FDG PET/CT before treatment showed multiple foci of abnormally increased glucose metabolism in the right upper and middle lobes of the lung, mediastinum, and right femur. (b, c) Images show a mass in the right lower and middle lobes of the lung (maximum transverse diameter approximately 5.6 cm) with abnormally increased glucose metabolism (SUVmax of 21.96, SUL of 17.58, MTV of 118.81 mm^3^, TLG of 939.79 g). The patient also presented with multiple mediastinal lymph node metastases and multiple metastases in the upper segment of the right femur (d, e), all of which showed abnormally increased glucose metabolism (WMTV of 191.06 mm3, WTLG of 13,804.09 g). Prior to treatment, 20 CTCs were detected in the patient's peripheral blood, including 4 PD‐L1+ epithelial CTCs (f), 10 PD‐L1‐ epithelial CTCs (g), and 6 PD‐L1‐ mixed‐type CTCs (h), with representative examples shown in the figures. The patient's PFS was 5.7 months, and OS was 11 months.

**Table S1:** Correlation between PD‐L1 expression in primary tumor tissue and PET/CT metabolic parameters.
**Table S2:** Metabolic parameters and CTCs for primary lung tumor by their immunohistochemistry PD‐L1 TPS groups.
**Table S3:** Metabolic parameters and CTCs for primary lung tumor by PD‐L1 TPS above or below the 1% threshold for positive expression.
**Table S4:** Metabolic parameters and CTCs for primary lung tumor by PD‐L1 TPS above or below the 50% threshold for positive expression.

## Data Availability

The datasets generated during and/or analyzed during the current study are not publicly available, but are available from the corresponding author on reasonable request.
