# Supplementary material for: Prognostic and Predict Value of Peripheral Blood Circulating Tumor Cells Programmed Death‐Ligand 1 Expression and F‐18‐Fluorodeoxyglucose Metabolic Parameters in Patients With Advanced Non‐Small Cell Lung Cancer Treated With Immune Checkpoint Inhibitors
Source: Cancer Med. 2026 Jun 10;15(6):e72001. doi: 10.1002/cam4.72001 (PMC13250698; doi:10.1002/cam4.72001)
Supplement: Supplementary file 1 — Figure S1: Comparative analysis of the percentage of PD‐L1 expression in tumor tissue and CTCs of matched patients with advanced NSCLC. (a) In all patients (n = 42), percentage of PD‐L1 expression in tumor tissue was significantly lower than the percentage of PD‐L1+ CTCs (median: 0.5% vs. 56%; p < 0.001); (b) In patients with TPS < 50% (n = 35), percentage of PD‐L1 expression in tumor tissue was significantly lower than the percentage of PD‐L1+ CTCs (median 0% vs. 56%, p<0.001). (c) In patients with TPS ≥ 50% (n = 7), TPS was significantly higher than the percentage of PD‐L1+ CTCs (median 85% vs. 38%, p = 0.016). **p < 0.01, ***p < 0.001. Figure S2: Correlation between the percentage of PD‐L1+ in tumor tissue and PD‐L1+ CTC. (a) In patients with PD‐L1+ CTC count ≥ 1/5 mL, there is no correlation between the percentage of PD‐L1+ CTC and the percentage of PD‐L1+ in tumor tissue (r = 0.03, p = 0.87). (b) In patients with PD‐L1+ CTC count ≥ 3/5 mL, there is no correlation between the percentage of PD‐L1+ CTCs and the percentage of PD‐L1+ in tumor tissue (r = 0.041, p = 0.827). (c) In NSCLC patients with TPS < 50%, there is no correlation between the percentage of PD‐L1 in tumor tissue and the percentage of PD‐L1+ CTC (r = 0.023, p = 0.885). (d) In NSCLC patients with TPS ≥ 50%, there is no correlation between the percentage of PD‐L1 in tumor tissue and the percentage of PD‐L1+ CTC (r = 0.152, p = 0.745). Figure S3: Correlation between PD‐L1 expression and different Subtypes of CTCs. Red indicates positive correlation, and blue indicates negative correlation. Among PD‐L1+ CTCs, mixed CTCs and mesenchymal CTCs showed a positive correlation with PD‐L1 expression (r = 0.35, p = 0.024 and r = 0.59, p < 0.001), with the correlation between mesenchymal CTCs and PD‐L1 expression being more significant. Figure S4: Tumor early response evaluation at 8 weeks based on RECISTv1.1 in NSCLC patients receiving ICI monotherapy or combined chemotherapy: differences between the early tumo [file CAM4-15-e72001-s001.doc]

Supplementary Figure 1 Comparative analysis of the percentage of PD-L1 expression in tumor tissue and CTCs of matched patients with advanced NSCLC. (a) In all patients(n=42), percentage of PD-L1 expression in tumor tissue was significantly lower than the percentage of PD-L1+ CTCs (median: 0.5% vs 56%; *p* <0.001);(b) In patients with TPS <50% (n=35), percentage of PD-L1 expression in tumor tissue was significantly lower than the percentage of PD-L1+ CTCs (median 0% vs. 56%, P＜0.001). (c) In patients with TPS ≥50% (n=7), TPS was significantly higher than the percentage of PD-L1+ CTCs (median 85% vs. 38%, P=0.016). **P < 0.01, ***P < 0.001

Supplementary Figure 2 Correlation between the percentage of PD-L1+ in tumor tissue and PD-L1+ CTC. (a) In patients with PD-L1+ CTC count ≥1/5ml, there is no correlation between the percentage of PD-L1+ CTC and the percentage of PD-L1+ in tumor tissue(r = 0.03, P = 0.87). (b) In patients with PD-L1+ CTC count ≥ 3/5ml, there is no correlation between the percentage of PD-L1+ CTCs and the percentage of PD-L1+ in tumor tissue (r = 0.041, P = 0.827). (c) In NSCLC patients with TPS＜50%, there is no correlation between the percentage of PD-L1 in tumor tissue and the percentage of PD-L1+ CTC (r=0.023, P=0.885）. (d) In NSCLC patients with TPS≥50%, there is no correlation between the percentage of PD-L1 in tumor tissue and the percentage of PD-L1+ CTC (r=0.152, P=0.745).

Supplementary Figure 3 Correlation between PD-L1 expression and different Subtypes of CTCs. Red indicates positive correlation, and blue indicates negative correlation. Among PD-L1+ CTCs, mixed CTCs and mesenchymal CTCs showed a positive correlation with PD-L1 expression (r = 0.35, P = 0.024 and r = 0.59, P < 0.001), with the correlation between mesenchymal CTCs and PD-L1 expression being more significant.

Supplementary Figure 4 Tumor early response evaluation at 8 weeks based on RECISTv1.1 in NSCLC patients receiving ICI monotherapy or combined chemotherapy: differences between the early tumor response group and the no response group. (a) Patients exhibiting early tumor response had lower baseline PD-L1+ mixed CTCs than patients with no early tumor response. (b-c) Patients exhibiting early tumor response had higher SUVmax and SUL values than patients with no early tumor response. *P < 0.05, **P < 0.01.


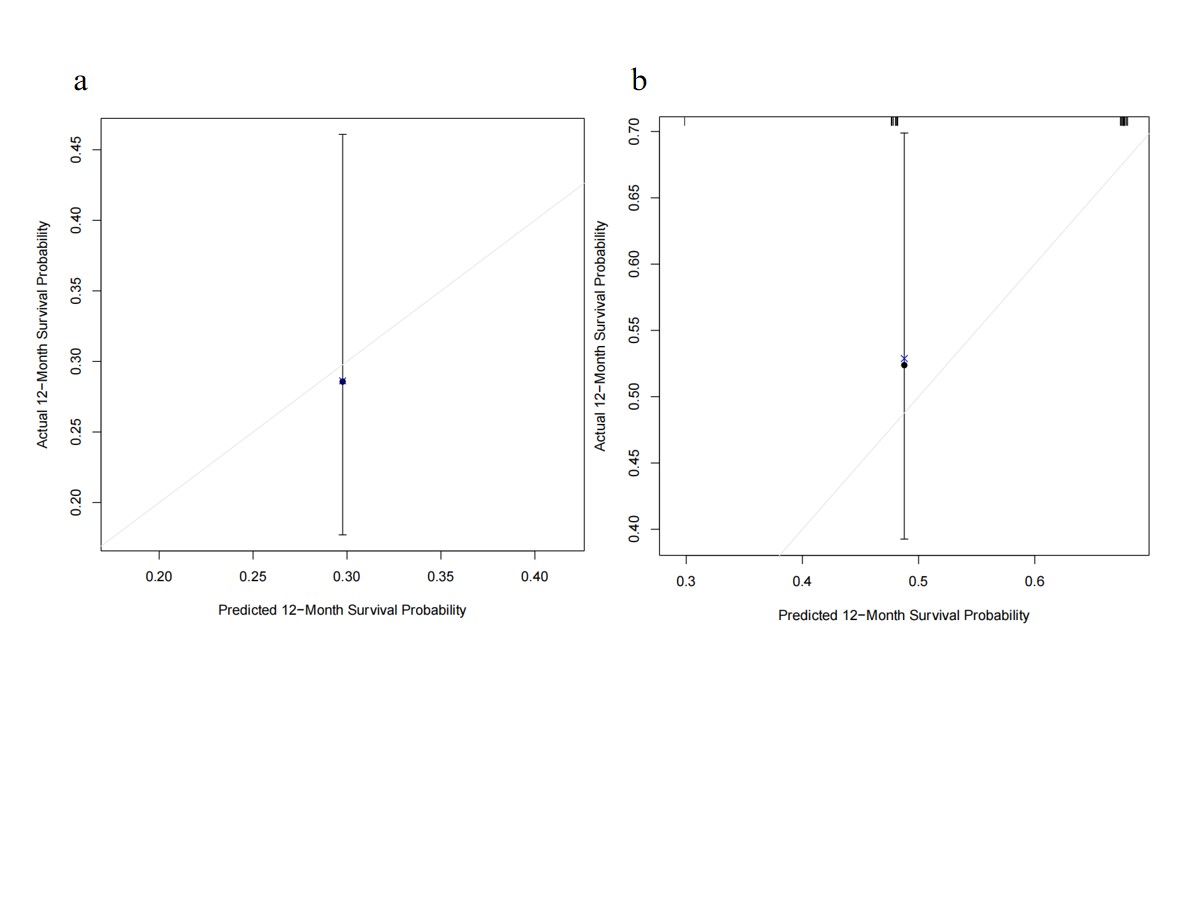


Supplementary Figure 5 **Calibration curves for WTLG and PD-L1+ mesenchymal CTCs in predicting progression-free survival (PFS) (a), and for PD-L1+ mesenchymal CTCs in predicting overall survival (OS)(b).** The plots demonstrate the calibration performance of the models, indicating agreement between predicted probabilities and observed outcomes. Blue data points cluster closely along the 45-degree diagonal line (ideal calibration line), indicating good calibration of the Cox regression models for PFS and OS prediction. Predicted risks align well with actual events, with no evidence of systematic overestimation or underestimation of risk (Bootstrap resampling: B=1000).


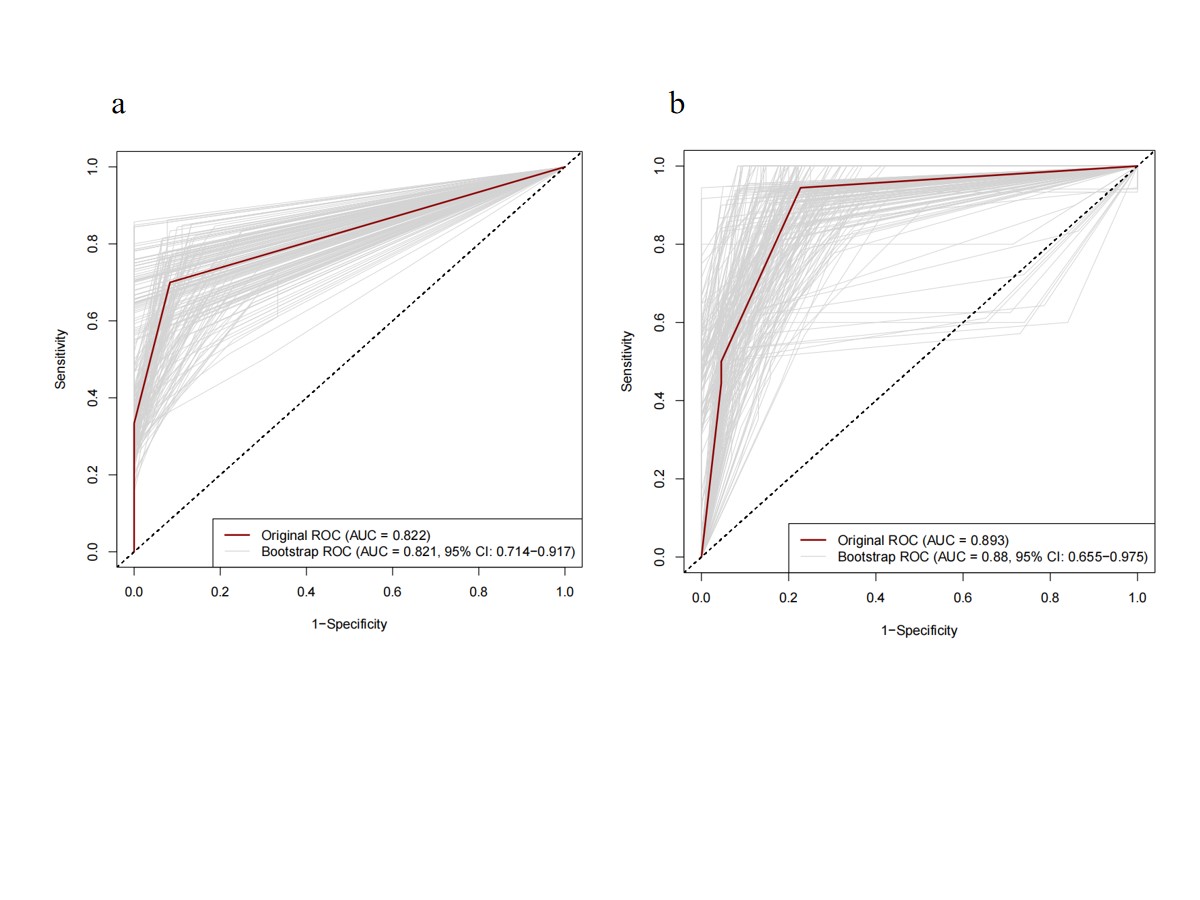


Supplementary Figure 6 **ROC curves for WTLG and PD-L1+ mesenchymal CTCs in predicting progression-free survival (PFS) (a), and for PD-L1+ mesenchymal CTCs in predicting overall survival (OS)(b).** The original ROC curves (solid red lines) yielded AUC values of 0.822 (a) and 0.893 (b). Following 1000 bootstrap resampling validations (thin gray lines), the mean AUCs were 0.821 (95% CI: 0.714–0.917) for (a) and 0.880 (95% CI: 0.655–0.975) for (b), demonstrating stable and robust predictive performance with preserved high discriminative power after internal validation.


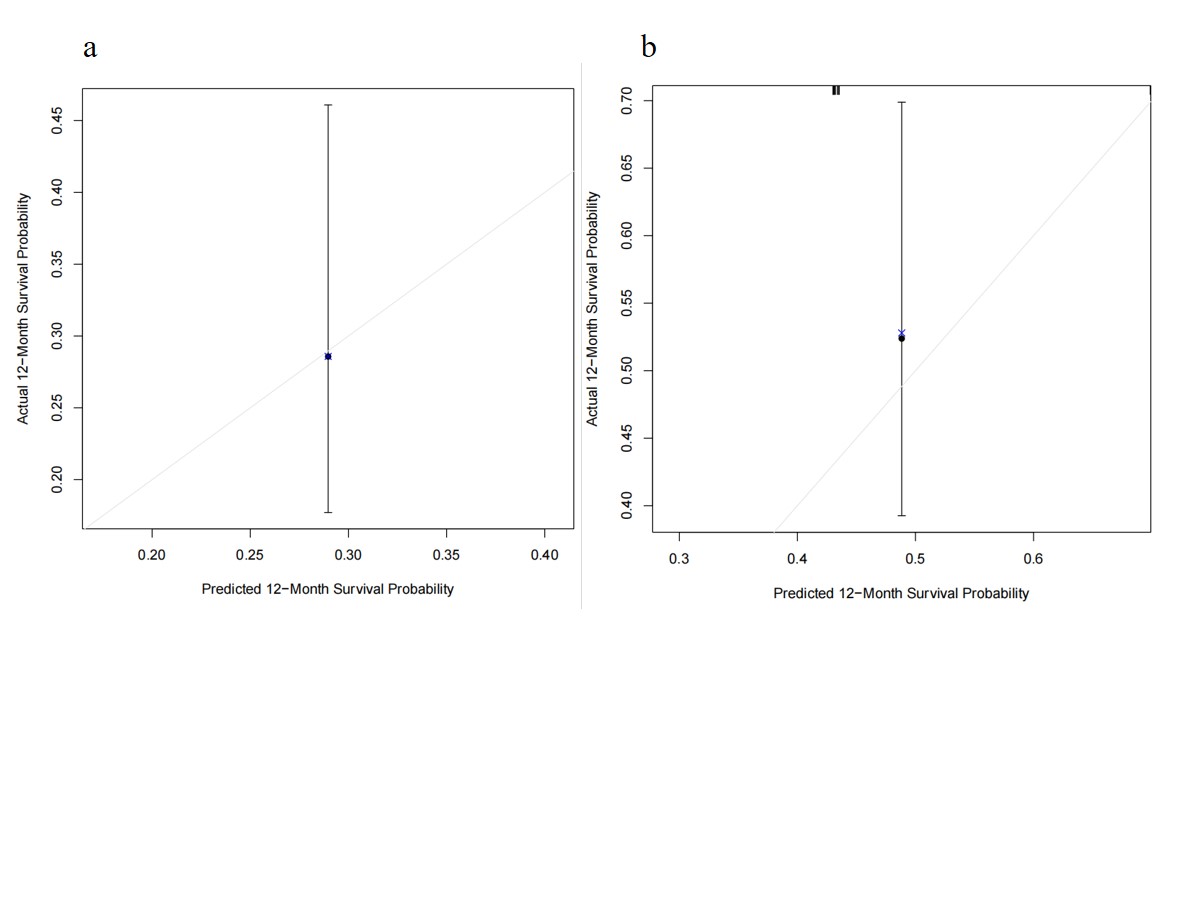


Supplementary Figure 7 **Calibration curves for WTLG combine with PD-L1+ mesenchymal CTCs in predicting progression-free survival (PFS)(a) and overall survival (OS)(b).** The plots demonstrate the calibration performance of the models, indicating agreement between predicted probabilities and observed outcomes. Blue data points cluster closely along the 45-degree diagonal line (ideal calibration line), indicating good calibration of the Cox regression models for PFS and OS prediction. Predicted risks align well with actual events, with no evidence of systematic overestimation or underestimation of risk (Bootstrap resampling: B=1000).


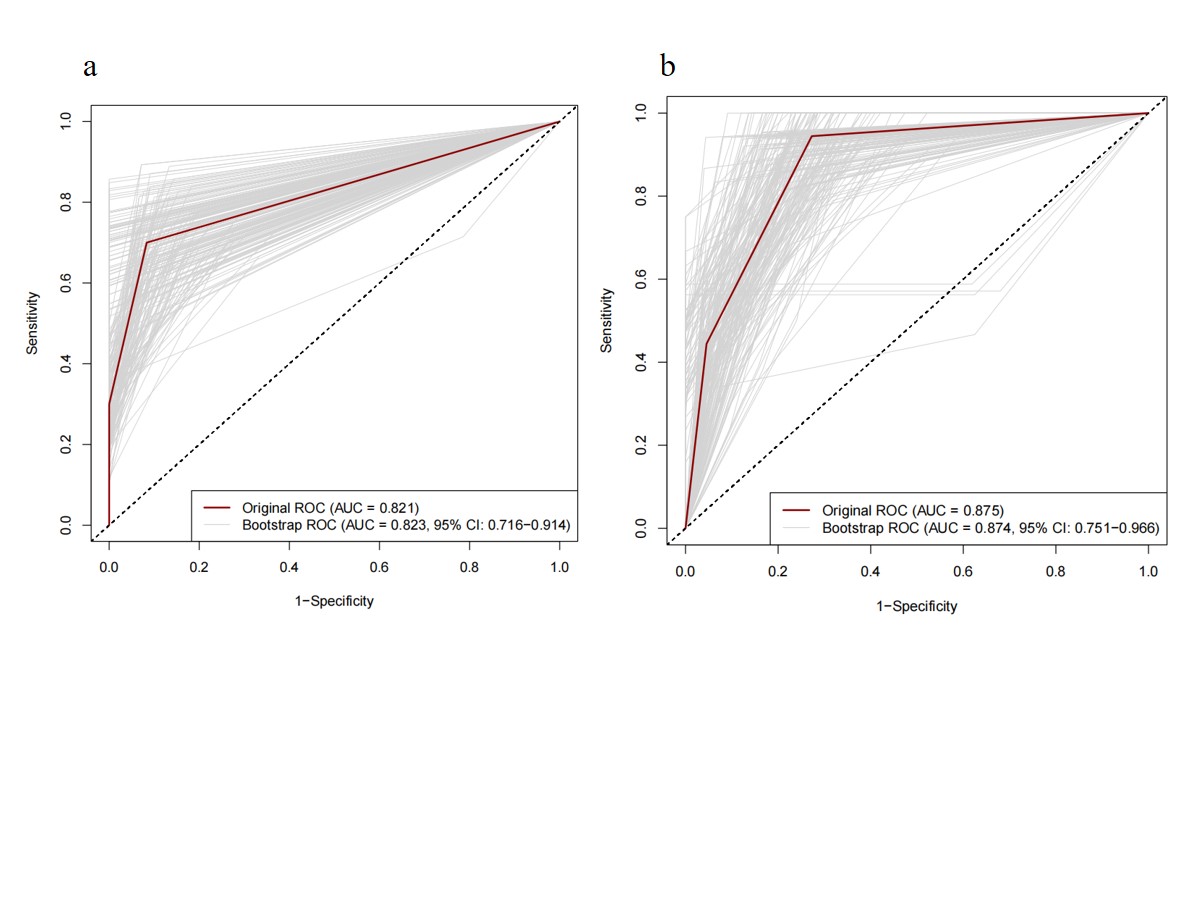


Supplementary Figure 8 **ROC curves for WTLG combine with PD-L1+ mesenchymal CTCs in predicting progression-free survival (PFS)(a) and overall survival (OS)(b).** The original ROC curves (solid red lines) yielded AUC values of 0.821 (a) and 0.875 (b). Following 1000 bootstrap resampling validations (thin gray lines), the mean AUCs were 0.823 (95% CI: 0.716–0.914) for (a) and 0.875 (95% CI: 0.751–0.966) for (b), demonstrating stable and robust predictive performance with preserved high discriminative power after internal validation.


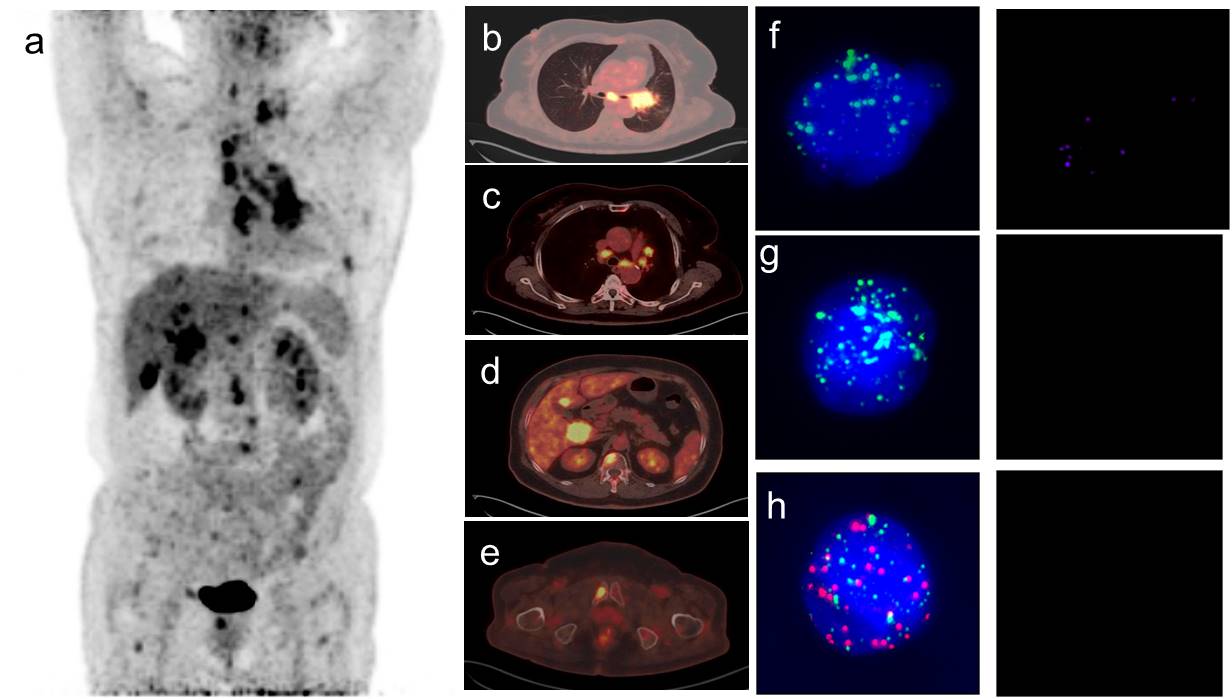


Supplementary Figure 9 18F-FDG PET/CT and CTC images of a patient with stage IV NSCLC. The patient was a 69-year-old female with adenocarcinoma, treated with chemoimmunotherapy. (a) The 18F-FDG PET/CT MIP image before treatment shows multiple foci of abnormally increased glucose metabolism throughout the body. (b) A mass in the upper lobe of the left lung (maximum transverse diameter approximately 4.75cm) with abnormally increased glucose metabolism (SUVmax of 8.68, SUL of 6.83, MTV of 50.52 mm3, TLG of 219.76 g). The patient also had multiple metastases in the mediastinal lymph nodes (c), liver (d), and right pubic bone (e), all of which showed abnormally increased glucose metabolism (WMTV of 243.23 mm3, WTLG of 46872.85 g). In addition, 25 CTCs were detected in the patient's peripheral blood before treatment, including 8 PD-L1+ mesenchymal CTCs (f), 7 PD-L1- mesenchymal CTCs (g), and 10 PD-L1- mixed CTCs (h), typical examples of which are shown in the figure. The patient's PFS was 2.0 months, and OS was 4.5 months.


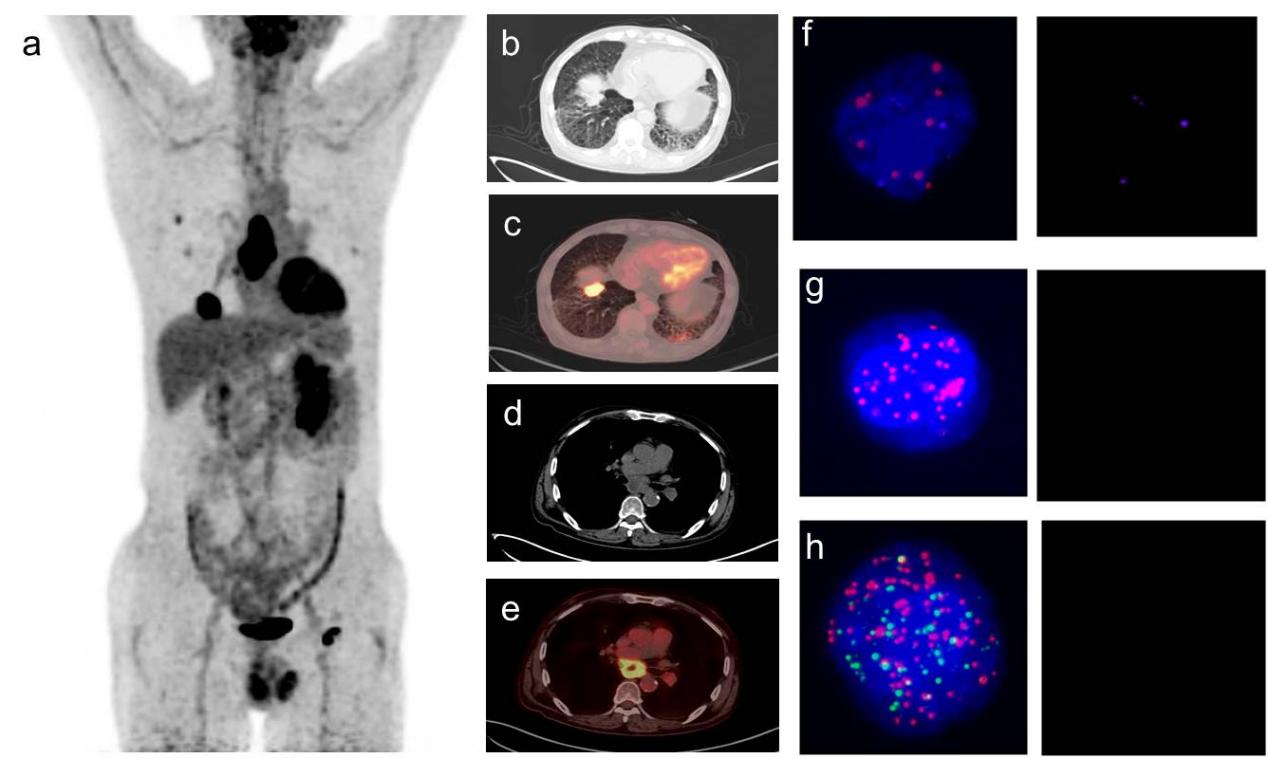


Supplementary Figure 10 18F-FDG PET/CT and CTC images of a patient with stage III NSCLC. The patient was a 59-year-old male with squamous cell carcinoma, treated with immunotherapy monotherapy. (a) Pre-treatment 18F-FDG PET/CT MIP image demonstrates abnormally increased glucose metabolism in the right lower lobe and mediastinum. (b-c) Images show a nodule in the right lower lobe (maximum diameter approximately 3.0cm) with abnormally increased glucose metabolism (SUVmax 14.18, SUL 12.00, MTV 26.18 mm3, TLG 96.13g). The patient also presented with mediastinal (subcarinal) lymph node metastasis (d-e), and all metastatic lesions showed abnormally increased glucose metabolism (WMTV 120.13mm3, WTLG 528.07g). A total of 8 CTCs were detected in the patient's peripheral blood prior to treatment, including 1 PD-L1+ epithelial CTC (f), 5 PD-L1- epithelial CTCs (g), and 2 PD-L1- mixed-type CTCs (h), with typical examples shown in the figure. As of the follow-up date, the patient was alive and had not experienced progression.


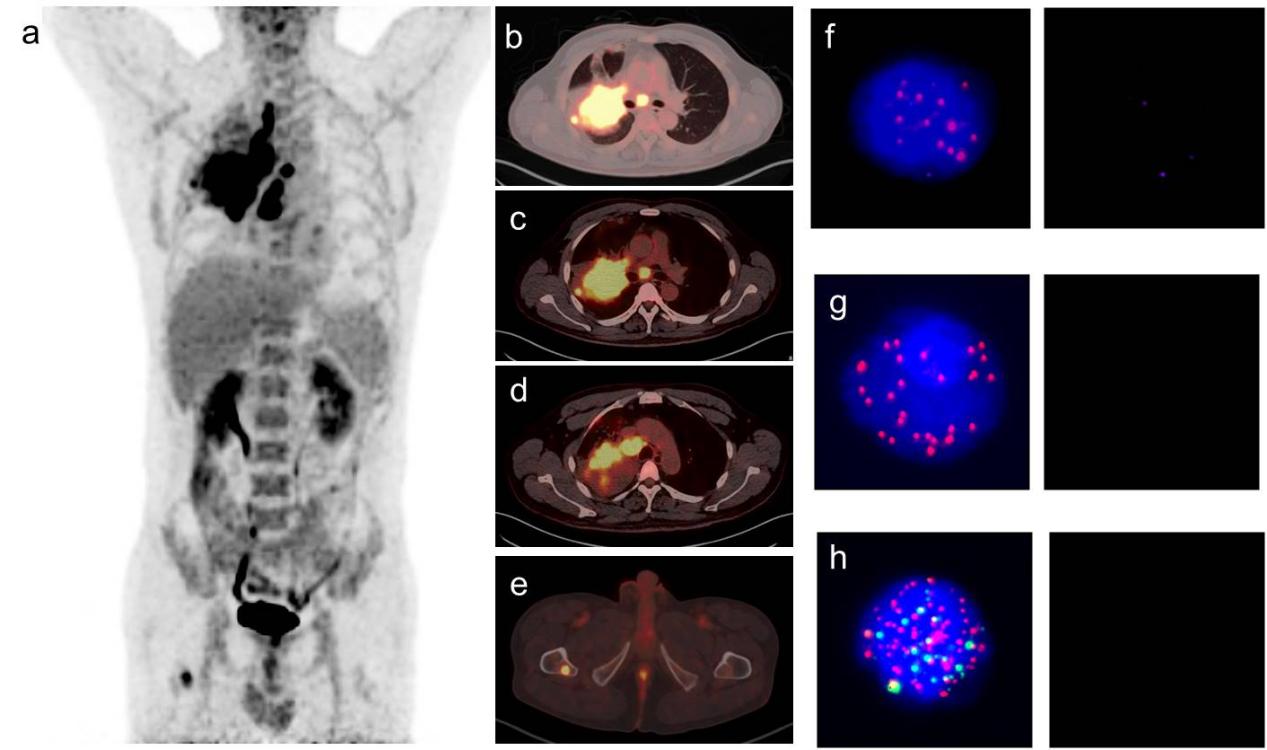


Supplementary Figure 11 18F-FDG PET/CT and CTC images of a patient with stage IV NSCLC. The patient was a 47-year-old male with adenocarcinoma, treated with immunotherapy combined with chemotherapy. (a) The MIP image of 18F-FDG PET/CT before treatment showed multiple foci of abnormally increased glucose metabolism in the right upper and middle lobes of the lung, mediastinum, and right femur. (b-c) Images show a mass in the right lower and middle lobes of the lung (maximum transverse diameter approximately 5.6cm) with abnormally increased glucose metabolism (SUVmax of 21.96, SUL of 17.58, MTV of 118.81mm3, TLG of 939.79g). The patient also presented with multiple mediastinal lymph node metastases and multiple metastases in the upper segment of the right femur (d-e), all of which showed abnormally increased glucose metabolism (WMTV of 191.06mm3, WTLG of 13804.09g). Prior to treatment, 20 CTCs were detected in the patient's peripheral blood, including 4 PD-L1+ epithelial CTCs (f), 10 PD-L1- epithelial CTCs (g), and 6 PD-L1- mixed-type CTCs (h), with representative examples shown in the figures. The patient's PFS was 5.7 months, and OS was 11 months.
