# Supplementary material for: Prognostic and Predict Value of Peripheral Blood Circulating Tumor Cells Programmed Death‐Ligand 1 Expression and F‐18‐Fluorodeoxyglucose Metabolic Parameters in Patients With Advanced Non‐Small Cell Lung Cancer Treated With Immune Checkpoint Inhibitors
Source: Cancer Med. 2026 Jun 10;15(6):e72001. doi: 10.1002/cam4.72001 (PMC13250698; doi:10.1002/cam4.72001)
Supplement: Supplementary file 2 — Table S1: Correlation between PD‐L1 expression in primary tumor tissue and PET/CT metabolic parameters. Table S2: Metabolic parameters and CTCs for primary lung tumor by their immunohistochemistry PD‐L1 TPS groups. Table S3: Metabolic parameters and CTCs for primary lung tumor by PD‐L1 TPS above or below the 1% threshold for positive expression. Table S4: Metabolic parameters and CTCs for primary lung tumor by PD‐L1 TPS above or below the 50% threshold for positive expression. [file CAM4-15-e72001-s002.doc]

Supplementary Table 1 Correlation between PD-L1 expression in primary tumor tissue and PET/CT metabolic parameters

| Factors | Spearman’s rho(r) | 95%CI | P value |
| --- | --- | --- | --- |
| SUVmax | 0.684 | 0.472-0.821 | ＜0.001 |
| SUL | 0.603 | 0.358-0.770 | ＜0.001 |
| MTV | 0.243 | -0.075-0.516 | 0.121 |
| WMTV | 0.133 | -0.187-0.428 | 0.399 |
| TLG | 0.299 | -0.014-0.559 | 0.054 |
| WTLG | 0.089 | -0.230-0.390 | 0.575 |

Supplementary Table 2 Metabolic parameters and CTCs for primary lung tumour by their immunohistochemistry PD-L1 TPS groups

| Factors | TPS＜1%（n=21） | 1%≤TP＜50%  （n=14） | | TPS≥50%  （n=7） | P value |
| --- | --- | --- | --- | --- | --- |
| SUVmax | 15.84±6.42（3.86-30.46） | | 18.63±5.79（6.38-34.50） | 26.84±5.64（17.48-33.30） | ＜0.001^a^ |
| SUL | 12.35±4.95（3.50-23.40） | | 14.13±4.67（5.66-25.70） | 19.99±4.17（11.50-22.00） | ＜0.001^a^ |
| MTV (mm^3^) | 95.4（17.00-654.59） | | 91.22（10.95-471.26） | 137.34（8.56-342.50） | 0.699^b^ |
| WMTV (mm^3^) | 263.54（30.98-3876.24） | | 171.96（66.87-929.26） | 238.05（78.49-734.36） | 0.441^b^ |
| TLG (g) | 386.46（61.27-3685.34） | | 624.86（20.43-4693.75） | 1073.89（31.48-1873.20） | 0.660^b^ |
| WTLG(g) | 5487.52（104.65-456451.3） | | 1122.61（186.79-425601.08） | 1664.02（309.18-107667.07） | 0.481^b^ |
| PD-L1+CTCs/5ml | 4（1-21） | | 4（0-25） | 6（1-20） | 0.265^b^ |
| PD-L1+Epithelial CTCs/5ml | 2（0-4） | | 2（0-4） | 2（0-8） | 0.643^b^ |
| PD-L1+Mixed CTCs/5ml | 1（0-4） | | 1（0-5） | 0（0-3） | 0.590^b^ |
| PD-L1+ Mesenchymal CTCs/5ml | 0（0-20） | | 0（0-20） | 0（0-10） | 0.599^b^ |
| PD-L1+CTC(%) | 50%（33-100%） | | 58%（0-100%） | 53%（20-80%） | 0.769^c^ |

a = One-way ANOVA test; b = Non-parametric rank-sum test; c = Pearson's chi-squared test

Supplementary Table 3 Metabolic parameters and CTCs for primary lung tumour by PD-L1 TPS above or below the 1% threshold for positive expression.

| Factors | TPS＜1%  （n=21） | TPS≥1%  （n=21） | P value |
| --- | --- | --- | --- |
| SUVmax | 16.33±6.60（3.86-30.46） | 21.91±7.09（6.38-34.50） | 0.012^a^ |
| SUL | 12.83±5.25（3.50-23.04） | 16.36±5.28（5.65-25.70） | 0.037^a^ |
| MTV (mm^3^) | 95.40（17.00-654.59） | 106.51（8.56-471.26） | 0.677^b^ |
| WMTV (mm^3^) | 263.54（30.98-3876.24） | 208.16（66.87-929.26） | 0.356^b^ |
| TLG (g) | 386.46（61.27-3685.34） | 849.14（20.43-4693.75） | 0.587^b^ |
| WTLG(g) | 5487.52（104.65-456451.30） | 1409.13（186.79-425414.08） | 0.318^b^ |
| PD-L1+CTCs/5ml | 4（1-21） | 5（0-25） | 0.929^b^ |
| PD-L1+Epithelial CTCs/5ml | 2（0-4） | 2（0-8） | 0.826^b^ |
| PD-L1+Mixed CTCs/5ml | 1（0-4） | 0（0-5） | 0.747^b^ |
| PD-L1+ Mesenchymal CTCs/5ml | 0（0-20） | 0（0-20） | 0.852^b^ |
| PD-L1+CTC(%) | 50%（33-100%） | 56%（0-100%） | 0.809^c^ |

a = Independent samples t-test; b = Non-parametric rank-sum test; c = Chi-squared test

Supplementary Table 4 Metabolic parameters and CTCs for primary lung tumour by PD-L1 TPS above or below the 50% threshold for positive expression

| Factors | TPS＜50%  （n=35） | TPS≥50%  （n=7） | P value |
| --- | --- | --- | --- |
| SUVmax | 18.09±7.11（3.86-34.50） | 24.91±5.93（17.48-33.30） | 0.016^a^ |
| SUL | 13.97±5.58  （3.50-25.70） | 18.12±3.73（11.50-22.00） | 0.054^a^ |
| MTV (mm^3^) | 93.31（10.95-654.59） | 137.34（8.56-342.50） | 0.958^b^ |
| WMTV (mm^3^) | 253.39（30.98-3876.24） | 238.05（78.49-743.36） | 0.725^b^ |
| TLG (g) | 522.13（20.43-4693.75） | 1073.89（31.48-1873.72） | 0.888^b^ |
| WTLG(g) | 1529.42（104.65-456346.65） | 1664.02（309.18-107667.07） | 0.962^b^ |
| PD-L1+CTCs/5ml | 4（0-25） | 6（1-20） | 0.320^b^ |
| PD-L1+Epithelial CTCs/5ml | 2（0-4） | 2（0-8） | 0.440^b^ |
| PD-L1+Mixed CTCs/5ml | 1（0-5） | 0（0-3） | 0.130^b^ |
| PD-L1+ Mesenchymal CTCs/5ml | 0（0-20） | 0（0-10） | 0.498^b^ |
| PD-L1+CTC(%) | 57%（0-100%） | 53%（20-80%） | 0.368^c^ |

a = Independent samples t-test; b = Non-parametric rank-sum test; c = Chi-squared test
